# Invasion, isolation and evolution shape population genetic structure in *Campanula rotundifolia*

**DOI:** 10.1093/aobpla/plaa011

**Published:** 2020-03-12

**Authors:** Julia Wilson, Annika Perry, Jessica R Shepherd, Mario Durán-Castillo, Christopher E Jeffree, Stephen Cavers

**Affiliations:** 1 UK Centre for Ecology & Hydrology, Penicuik, Midlothian, Scotland, UK; 2 Royal Botanic Garden Edinburgh, Edinburgh, Scotland, UK; 3 Instituto Nacional de Investigaciones Forestales, Agrícolas y Pecuarias (INIFAP), Campeche, Mexico; 4 Institute of Molecular Plant Sciences, Daniel Rutherford Building, The King’s Buildings, University of Edinburgh, Edinburgh, Scotland, UK

**Keywords:** Allopatric, *Campanula rotundifolia*, chloroplast DNA, common garden, cytotype, genome size, minority cytotype, phenology, polyploidy, reproductive barrier, sympatric

## Abstract

The distribution and genetic structure of most plant species in Britain and Ireland bear the imprint of the last ice age. These patterns were largely shaped by random processes during recolonization but, in angiosperms, whole-genome duplication may also have been important. We investigate the distribution of cytotypes of *Campanula rotundifolia*, considering DNA variation, postglacial colonization, environmental partitioning and reproductive barriers. Cytotypes and genome size variation from across the species’ range were determined by flow cytometry and genetic variation was assessed using cpDNA markers. A common garden study examined growth and flowering phenology of tetraploid, pentaploid and hexaploid cytotypes and simulated a contact zone for investigation of reproductive barriers. Irish populations were entirely hexaploid. In Britain, hexaploids occurred mostly in western coastal populations which were allopatric with tetraploids, and in occasional sympatric inland populations. Chloroplast markers resolved distinct genetic groups, related to cytotype and geographically segregated; allopatric hexaploids were distinct from tetraploids, whereas sympatric hexaploids were not. Genome downsizing occurred between cytotypes. Progeny of open-pollinated clones from the contact zone showed that maternal tetraploids rarely produced progeny of other cytotypes, whereas the progeny of maternal hexaploids varied, with frequent pentaploids and aneuploids. The presence of distinctive hexaploid chloroplast types in Ireland, Scottish islands and western mainland Britain indicates that its establishment preceded separation of these land masses by sea-level rise *c.* 16 000 years BP. This group did not originate from British tetraploids and probably diverged before postglacial invasion from mainland Europe. The combination of cytotype, molecular, contact zone and common garden data shows an overall pattern reflecting postglacial colonization events, now maintained by geographic separation, together with more recent occasional local *in situ* polyploidisation. Reproductive barriers *favour the persistence of the tetraploid to the detriment of the hexaploid.*

## Teaser

*Campanula rotundifolia* has a distinctive distribution of tetraploid and hexaploid cytotypes in Britain and Ireland. Populations are mostly allopatric, with some exceptions. Chloroplast markers show that allopatric hexaploids are distinct from tetraploids, whereas sympatric hexaploids are not, and that allopatric hexaploids did not originate from British tetraploids. At a simulated contact zone, maternal tetraploids rarely produced progeny of other cytotypes, but maternal hexaploids produced many pentaploids and aneuploids. Present-day distribution reflects postglacial recolonization events, now maintained by geographic separation, together with more recent occasional local *in situ* polyploidisation. Reproductive barriers favour the persistence of the tetraploid over the hexaploid.

## Introduction

Genetic structure in British and Irish plant species has been enduringly shaped by events during and since the last glacial period (Cottrell *et al.* 2002; Rendell and Ennos 2002; Provan and Bennett 2008; Wachowiak *et al.* 2011). When the British–Irish ice sheet was at its maximum extent *c.* 27 000 years BP (during the British Devensian and Irish Midlandian stages), only parts of southern and eastern England remained unglaciated, and grounded ice reached to the edge of the continental shelf along its whole boundary from SW Ireland to the Shetland Isles (Clark *et al.* 2012). With sea levels around 130 m lower than at present, land bridges connected Britain and Ireland to each other and to continental Europe, and more southerly, non-glaciated parts of the continental shelf were dry land. A retreat of the ice sheet and rise in sea level followed, so that most of the land mass was deglaciated by 15 000 years BP, and the land bridges and continental shelf were progressively inundated. Ireland became an island at about this time (Clark *et al.* 2012) while Britain remained connected to Europe along a broad but gradually narrowing front along the English Channel and the southern North Sea for another 7000 years. The process of recolonization of deglaciated land resulted in distinctive genetic patterns, still evident today (Hewitt 1999), and offered opportunities for species with adaptive strategies, such as genome doubling, to take advantage of the new space.

Polyploidy (whole-genome duplication) is a widespread feature of angiosperms and the resulting changes in genetic and genomic attributes can be advantageous (Soltis and Soltis 2000). It plays an important role in plant invasions (te Beest *et al.* 2012) and the rapid evolution of new species (Ramsey and Schemske 1998), and many plant species consist of cryptic polyploid races (e.g. Soltis and Soltis 2000; Suda *et al.* 2007). Autopolyploidy is a route to speciation (Soltis *et al.* 2007) and formation of autopolyploid lineages may provide short- and long-term evolutionary advantages through polysomic inheritance and increased genome flexibility (Parisod *et al.* 2010). Whole-genome duplication is often accompanied by genome downsizing (Leitch and Bennett 2004), and analysis of patterns of genome size variation may provide insight to the evolutionary histories of different populations (Balao *et al.* 2009; Certner *et al.* 2017).

**Figure 1.**
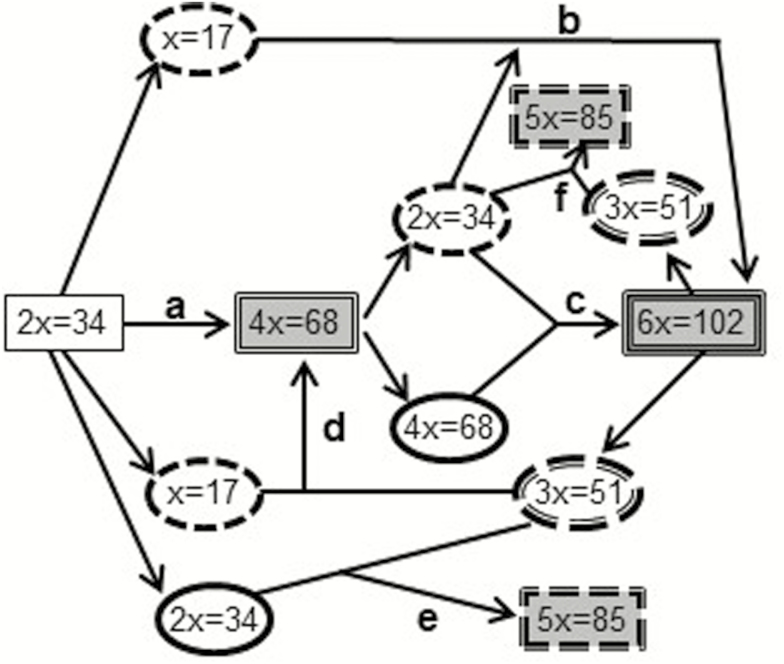
Potential routes to formation of polyploid *C. rotundifolia*. (a) Tetraploid formation by auto polyploidization of diploid, (b) hexaploid formation by backcross between tetraploid and diploid with subsequent polyploidization, (c) hexaploid formation via reduced and unreduced tetraploid gametes, (d) tetraploid formation by diploid × hexaploid cross, (e) pentaploid formation by hexaploid × diploid cross with diploid unreduced gametes, (f) pentaploid formation by tetraploid × hexaploid cross. Ellipses represent reduced (dotted margin) and unreduced (solid margin) gametes. Shaded symbols indicate cytotypes occurring in Britain and Ireland. For simplicity, the triploid bridge (Ramsey and Schemske 1998) (an alternative route to tetraploid formation from diploids) is omitted.

Polyploids can arise through a variety of pathways (Fig. 1). Tetraploids can result from autopolyploidization of diploids, or crosses between hexaploids and diploids. Hexaploids can arise from backcrossing between tetraploids and diploids with subsequent polyploidization, or from crosses of reduced and unreduced gametes from tetraploids. Once polyploids have arisen, their establishment requires escape from minority cytotype exclusion at contact zones by pre- or post-zygotic isolation from their progenitors. This can occur through divergence of habitat preferences, phenological characters or pollinator associations, or by reproductive barriers—producing sterile intermediates or the effect of the ‘triploid block’ (Petit *et al.* 1999).

While postglacial range expansion may have facilitated the emergence and survival of polyploids, the environmental conditions at the time of their expansion would have been completely different to those encountered today. Range expansion and consolidation must be accompanied by selection and adaptation to the prevailing climatic and environmental conditions, such processes continuing to the present. Contemporary environmental niches will not reflect those at the time of range expansion. Hence although deglaciation provides opportunities for polyploid invasion, present-day distribution of cytotypes may reflect both historical and contemporary factors. Historical factors relate to patterns of postglacial migration and founder effects (Hewitt 2000; Waters *et al.* 2013), such as those seen in the distribution of *Quercus* chloroplast haplotypes in European oaks (Cottrell *et al.* 2002; Petit *et al.* 2002) and *Hedera* nuclear and chloroplast haplotypes in European ivy (Grivet and Petit 2002). Contemporary factors include ecological requirements related to current climate—as in North American *Chamerion angustifolium* (Thompson *et al.* 2014), and Central European *Allium oleraceum* (Ježilová *et al.* 2015) and *Cardamine amara* (Zozomová-Lihová *et al.* 2015), reproductive isolation—as in *Aster amellus* (Castro *et al.* 2011) in Central Europe, or reproductive barriers and ecological differentiation—as in *Jacobaea carniolica* in Austria (Sonnleitner *et al.* 2013; Hülber *et al.* 2015).

During the late Devensian, a ‘cold-stage’ flora was present in British tundra regions south of the ice sheet. As conditions warmed, species moved north and gradually re-established on de-glaciated land, but movement from Europe and between Britain and Ireland became increasingly constrained by sea-level rise. This isolation led to the flora of Britain and Ireland being more limited than that of many European countries, and the Irish flora being more limited than that of Britain (Godwin 1975; Webb 1983). While many species are thought to have crossed into Ireland from Britain, molecular studies are yielding increasing evidence of a more direct migration route to Ireland from the south, across the Celtic Sea, for oak species (Kelleher *et al.* 2004) and components of the Irish ‘Lusitanian’ flora (Beatty and Provan 2013, 2014; Santiso *et al.* 2016).

### Study species

While little is known about the distribution of most polyploid species in Britain and Ireland, previous observations of cytotypes of *Campanula rotundifolia* (McAllister 1972; Stevens *et al.* 2012) indicate significant differences in their distribution within Britain and between Britain and Ireland and provide an opportunity to explore both the historical (postglacial invasion pathways) and contemporary factors (climate, reproductive isolation) influencing their distribution, which may also provide insights into the contrasting distributions of other species.

The herbaceous perennial *C. rotundifolia* is a species of wide ecological amplitude, metal-tolerant, insect-pollinated (by oligolectic and solitary bees) and largely self-incompatible. It has a circumpolar distribution in the northern hemisphere, forming a polyploid complex across Europe with a cytotype series of 2*n* = 34, 68, 85, 102 chromosomes (Stevens *et al.* 2012). Metaphase chromosomes (Laane *et al.* 1983) and isozyme analysis (Bingham and Ranker 2000) indicate that tetraploids (2*n* = 68) are autopolyploids.

**Figure 2.**
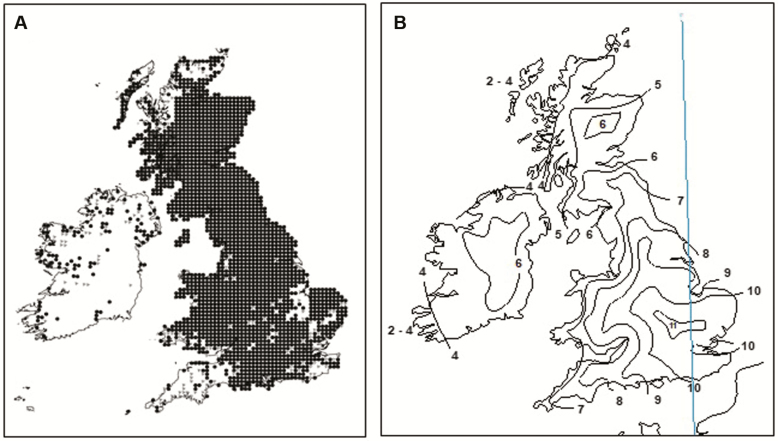
(A) The distribution of *Campanula rotundifolia* in Britain and Ireland. Each dot represents at least one record in a 10-km square of the National Grid, ● 1970 onwards, pre-1970. Reproduced by permission of the Botanical Society of Britain and Ireland. (B) Conrad’s continentality index (CCI) (Conrad, 1946) redrawn from Pyatt *et al.* (2001) for Britain, and Hargy (1997) for Ireland (the latter is reproduced by permission and © 1997 by the Royal Meteorological Society).

Both diploids and tetraploids are widespread in mainland Europe (Böcher 1960; Kovanda 1970; Laane *et al.* 1983) but hexaploids are rare, occurring occasionally in France (Kovanda 1970) and Spain (McAllister 1972). The species is widespread in Britain, where both hexaploids and tetraploids occur but only the hexaploid has been reported in Ireland, and diploids are absent in both (McAllister 1972; Stevens *et al.* 2012) (Fig. 2A). In Britain, the cytotypes are mostly allopatric: hexaploids have a predominantly westerly distribution, and tetraploids are more easterly. However, some populations are sympatric, and occasional pentaploids are present (McAllister 1972; Stevens *et al.* 2012). There are geographic and concomitant environmental components to their distributions; western and northern locations where hexaploids are common have more oceanic climates (Crawford 2000) (Fig. 2B), and higher rainfall (Crawford *et al.* 2003).

McAllister (1972) found little evidence of breeding barriers within or between tetraploid and hexaploid cytotypes, either of geographically similar or distant origins. Pentaploid offspring of crosses between British tetraploid and hexaploid cytotypes were vigorous, but their pollen production was often reduced. Pentaploids successfully backcrossed to both their parents and produced intermediate numbers of chromosomes in the F2 progeny. Sutherland and Galloway (2016) using accessions from Europe and North America in reciprocal diploid × tetraploid and tetraploid × hexaploid crosses showed that the former produced a mixture of triploid and tetraploid progeny, while the latter produced predominantly pentaploids, irrespective of which cytotype was maternal.

Divergence in flowering phenology may contribute to reproductive barriers. Long-term phenological data showed comparatively little regional variation (10 days) in the ordinal day of appearance of *C. rotundifolia* first flowers (first flowering date [FFD]) in Britain and Ireland, but plants in Ireland (presumably hexaploid) were the first to flower (Jeffree 1960). Elsewhere, data show later flowering at northerly latitudes (Bradley *et al.* 1999; Molau *et al.* 2005; Dunnell and Travers 2011). However, in comparative common garden studies, Turesson (1925, 1930) noted that alpine *C. rotundifolia* flowered earlier than lowland plants in southern Sweden, while Preite *et al.* (2015) found that flowering duration decreased with latitude and elevation in central Europe.

Sutherland and Galloway (2018a) constructed nuclear and chloroplast phylogenies of *C. rotundifolia* populations from Europe and North America, which included a single European hexaploid sample (from Ireland) and one tetraploid population from England in analysis of nuclear variation. The same two populations, together with a further population from England (mixed hexaploid/tetraploid) were also analysed for cpDNA variation. Differences between the two phylogenies led them to postulate different European glacial refugia for tetraploid and hexaploid populations.

The chloroplast genome is slowly evolving, effectively comprises a single locus and, in most angiosperms, reflects only the maternal lineage. These characteristics can be highly informative for studies of historical dispersal in seed-dispersed plant species and cpDNA data have consequently been widely used in phylogeography.

The objectives of this study were to evaluate the influence of both historical and contemporary factors on the present-day distribution of cytotypes in Great Britain and Ireland and to consider evidence relating to the species’ potential postglacial invasion pathways, especially in light of differences between the Irish and British flora. We used flow cytometry to determine the cytogeography and genome size of *C. rotundifolia*, network analysis of chloroplast DNA markers to understand the relationships between cytotypes, and supplemented this work with a common garden study (which simulated a contact zone) to investigate the reproductive and environmental mechanisms maintaining the contemporary distribution of cytotypes through studies of growth, flowering phenology and open-pollinated crosses. We integrate these multiple sources of evidence to understand the forces shaping the polyploid distribution and consider the stability of populations in light of these observations.

## Materials and Methods

Further details on collections, flow cytometry and molecular investigations are provided in **Supporting Information—Appendix S1**.

### Collections

More than 1700 samples of seeds, leaves or stem cuttings were collected and cytotyped **[see****Supporting Information—Table S1****]**. Most samples were from Britain and Ireland with additional seed samples from elsewhere in the species’ range. To avoid multiple sampling of clonal plants, samples were taken at least 5 m apart, from plants which were morphologically different. Previous observations had noted the occurrence of hexaploids outside their normal western range; at Wanlockhead in SW Scotland, Teesdale in central England and Cheddar Gorge in southern England (McAllister 1972; Stevens *et al.* 2012), and more intensive sampling was conducted in these areas to determine their extent. Some data are aggregated for presentation, forming ~700 locations, circumscribed by 0.01° × 0.01° latitude and longitude **[see****Supporting Information—Table S1****]**.

### Cytotype and genome size determination

**Figure 3.**
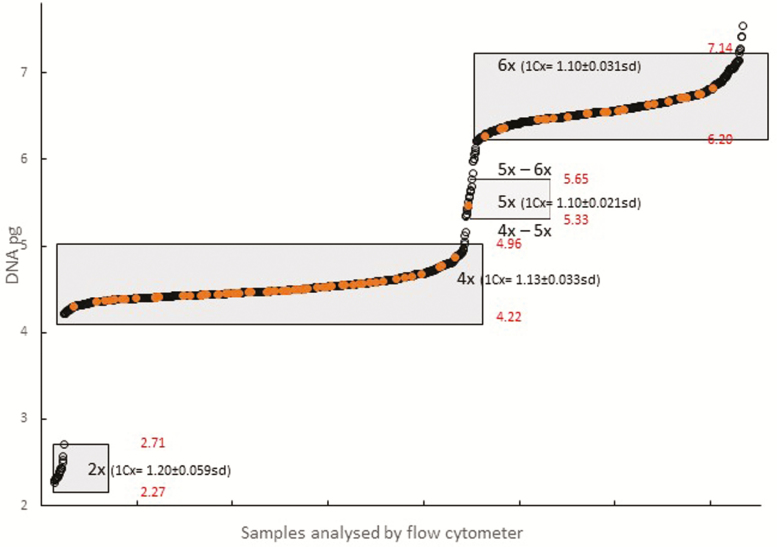
The distribution of genome size among 1664 field samples of *Campanula rotundifolia*. Samples measured by flow cytometry with internal reference standard of *Pisum sativum* Ctirad (9.09 pg). Samples for which chromosomes were also counted are indicated in orange. From this data set the mean 2C (±SD) pg DNA for cytotypes are 2.40 ± 0.11, 4.50 ± 0.13, 5.49 ± 0.11, 6.58 ± 0.19 pg DNA. Mean and standard deviation 1Cx values are shown for each cytotype. These samples were used to set pg size limits (shown in red) for definition of field collected cytotypes and hence the limits for aneuploids. These limits are applied in Figs 4 and 7, see **Supporting Information—Table S1**.

**Figure 4.**
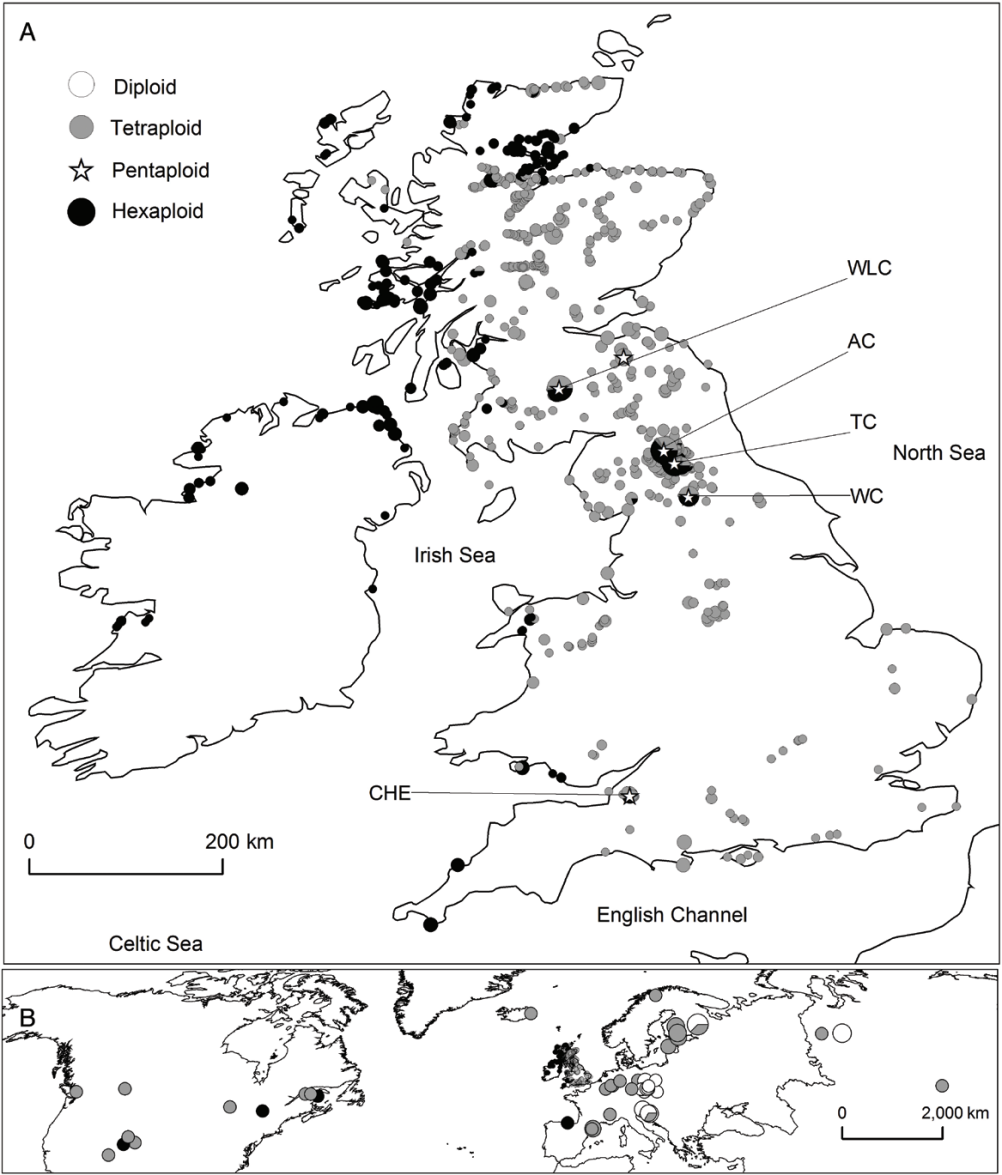
Distribution of cytotypes (A) in Britain and Ireland and (B) in the northern hemisphere. For readability, populations closer than 0.01° have been merged and the area of each circle (except those in Britain and Ireland in B) has been scaled according to the square root of the number of observations. Pentaploids are not scaled. Aneuploids not shown. Original data are in **Supporting Information—Table S1**. The smallest circles in each figure represent a single observation. Specific locations mentioned in the text are WLC (Wanlockhead/Leadhills cluster), AC (Alston cluster), TC (Teesdale cluster), WC (Wensleydale cluster) and CHE (Cheddar Gorge).

Cytotypes and genome size were determined by flow cytometry (Figs 3 and 4; **see****Supporting Information—Table S1**). Chromosome counts of 98 individuals **[see****Supporting Information—Table S1****]** were previously determined on root tips by cytological methods (Shepherd 2007). Their cytotype assignments were verified by flow cytometry in the current study and used to calibrate further flow cytometric data.

Sample preparation for flow cytometry followed the one-step protocol of Doležel *et al.* (2007), using an internal plant standard in each extract (*Pisum sativum* ‘Ctirad’, 2C = 9.09 pg DNA) (Doležel *et al.* 1998), propidium iodide (PI) dye and Tris·MgCl_2_ buffer. Tests demonstrated that DNA content of cytotypes might be affected by freshness of the sample, as also observed by Greilhuber *et al.* (2007) and Odee *et al.* (2015). Consequently, the DNA contents in Table 1 are restricted to those from samples analysed singly within 2 h of collection. Investigation of regional variation in Cx values focussed on samples from Britain and Ireland, in good condition **[see****Supporting Information—Appendix S1****]** and analysed within 1–3 days of collection.

**Table 1.** 2C and Cx DNA contents (pg) of fresh *C. rotundifolia* leaves from Europe and North America.

	Diploid	Tetraploid	Pentaploid	Hexaploid
Mean 2C (SD) DNA	2.40 (0.11)	4.51 (0.10)	5.56 (0.09)	6.53 (0.15)
Min	2.30	4.30	5.47	6.21
Max	2.71	4.89	5.65	6.89
Ratio mean DNA content to diploid	1	1.88	2.3	2.69
Mean Cx (SD) DNA	1.20 (0.056)	1.13 (0.025)	1.11 (0.018)	1.09 (0.025)
Number of observations	13	236	3	91

The full data set of all field samples, combining genome size from flow cytometry with chromosome counts, is shown in Fig. 3 which also defines the pg size limits which were set for different euploid cytotypes and aneuploid groups in the overall analysis.

### Molecular investigations

**Figure 5.**
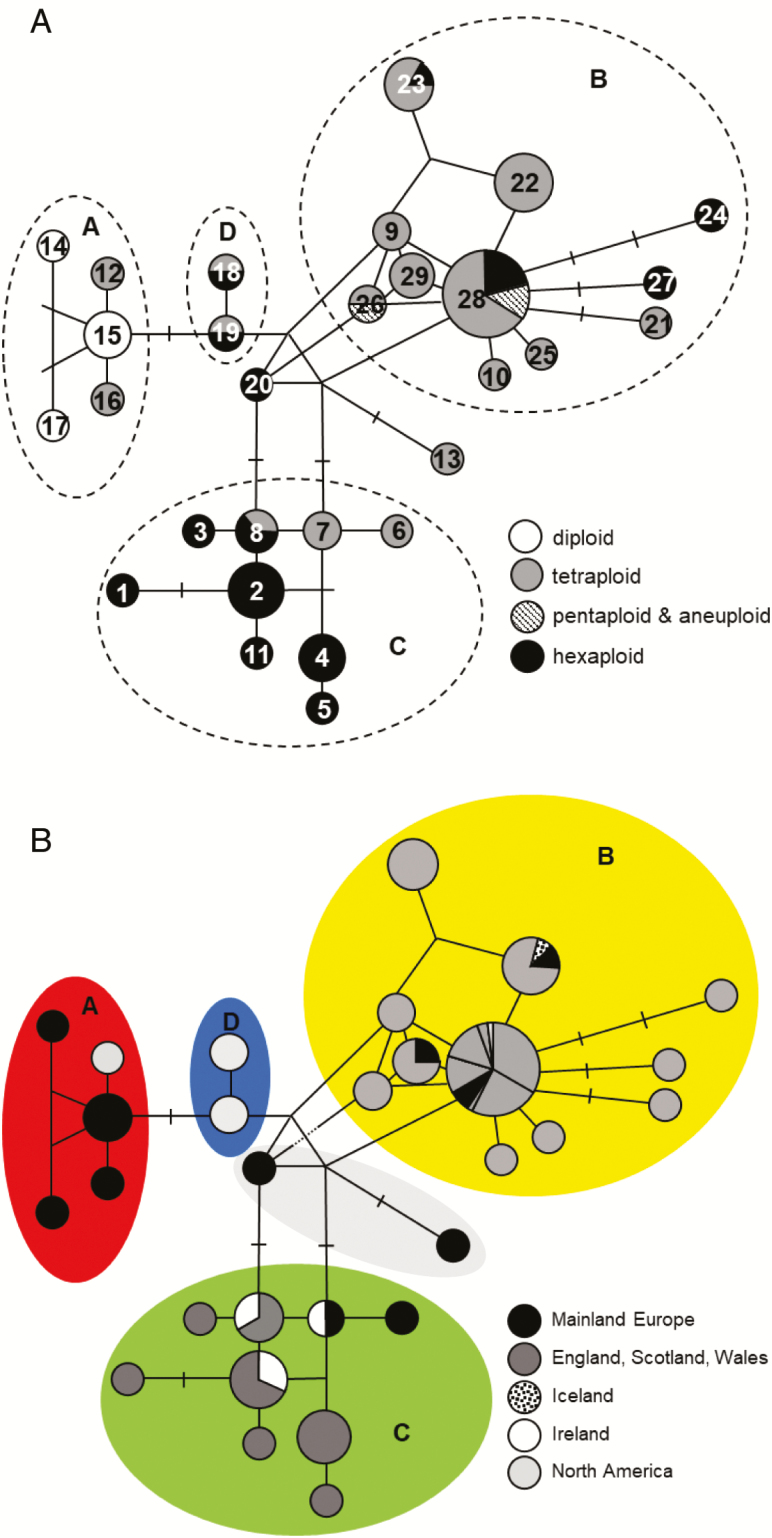
Minimum-spanning network of chloroplast DNA haplotypes of *Campanula rotundifolia* (A) haplotype by cytotype (B) haplotype by location. Haplotype numbers were arbitrarily assigned; links are one mutation except where indicated by ticks. Node sizes are proportional to frequencies, the smallest node corresponds to a single observation, the largest node corresponds to 53 observations. See also **Supporting Information—Tables S2****and****S3**. The colours in (B) provide the key for Fig. 6 and cross-reference to the colours in **Supporting Information—Tables S2****and****S3**.

Using universal primers (including those for microsatellites and other regions) a subset of samples was screened for variation in the chloroplast genome **[see****Supporting Information—Appendix S1****]**. Preliminary tests detected variation at several loci, and the markers *trn*C*-ycf*6, *trn*H*-trn*K and *trn*D*-trn*T were selected to screen 126 samples covering the full distribution of the collection **[see****Supporting Information—Table S1****]**. Chloroplast haplotypes were characterized based on these three loci **[see****Supporting Information—Table S2****]**, and minimum-spanning networks (Fig. 5) were constructed.

### Common garden study: growth, phenology and simulated contact zone

Clones for the common garden were produced in a glasshouse from cuttings and planted out in October 2008 at 0.3 × 0.4 m spacing into an external raised bed containing local topsoil mixed with 20 % grit. The site was in eastern Scotland (Midlothian) at 55.86°N, 3.21°W, 190 m above sea level (where the naturally occurring cytotype is tetraploid). It comprised five randomized blocks of clonal tetraploid, pentaploid and hexaploid *C. rotundifolia* covering the range of the species in Britain and Ireland **[see****Supporting Information—Table S1****]**. Each of the five blocks contained single replicates of 55 tetraploid, 1 pentaploid and 37 hexaploid clones.

#### ***Assessments***.

Plant growth and survival were monitored, and pollination was not restricted. Flowering phenology (FFD) (the ordinal date on which the first flower on a plant was sufficiently open to be entered by bees) was determined daily in 2009 and 2010. On 3 August 2009 (Day 215), the number of flowering stems per plant was counted, and three randomly selected flowering stems were taken from plants in blocks 1 and 3 and the number of open flowers, flower buds and swollen seed heads (indicating successful pollination) on these was determined **[see****Supporting Information—Fig. S1****]**. The above-ground parts of plants in blocks 2 and 5 were harvested (cut 5 cm above ground level) on 9 September 2009 (Day 252), dried and weighed.

This study simulated a reproductive contact zone between the three cytotypes of *C. rotundifolia* occurring in Britain and Ireland. In 2014 and 2015, seed (from 7 tetraploid, 1 pentaploid and 10 hexaploid clones) was germinated from the September 2009 harvest, and the cytotypes of the resultant seedling progeny determined by flow cytometry, using the size limits shown in Fig. 3 to allocate samples to euploids and aneuploids. All the seedlings tested possessed at least two post-cotyledonary leaves and appeared viable. Where possible, 30 seedlings per mother plant were analysed, but this was reduced in some cases due to poor germination.

The study site is surrounded by farmland, where the species is absent. The nearest plants are on hill tops 1.6 km westward, where populations are sparse and flower shoots are preferentially browsed by rabbits. Although data on pollinator species and their flight distances are not available for this site, Gathmann and Tscharntke (2002) report a flight distance of only 200 m for one such species. Although long distance pollination cannot be ruled out, it is most likely to have occurred preferentially between plants growing within the common garden, where the opportunities for cross-pollination with different cytotypes could influence the results. Through the harvest conducted on Day 215 in 2009 **[see****Supporting Information—Fig. S1****]**, the proportion of open flowers of the different cytotypes over the flowering season was estimated, and the observed and expected proportions of progeny of the maternal cytoype arising from freely outcrossing mother plants of tetraploid and hexaploid clones were calculated **[see****Supporting Information—Table S4****]**.

### Home site characterization

Conrad’s continentality index (CCI—a measure of mean annual temperature range) (Conrad 1946) (Fig. 2B) was determined for the home location of each clone and for the common garden site using the Ecological Site Classification Decision Support Tool (Pyatt *et al.* 2001) for sites in Britain, and Hargy (1997) for Ireland. Low values of CCI indicate oceanic climates with a narrow annual temperature range (mild winters, cool summers), and high values indicate more continental climates with a wider annual temperature range. Air temperature data from an adjacent meteorological site (Easter Bush) were used to calculate accumulated growing degree days (GDD) > 5 °C between 1 January in each year and the mean FFD of each clone. 

### Data analysis

The minimum-spanning network of chloroplast haplotypes was constructed using Network 4.6 (fluxus-engineering.com). One-way ANOVA (Genstat 16.1) with randomized block design was used to examine the relationships between FFD, home climate (CCI) and cytoype. Cytotype and CCI were used as separate factors. Differences between means were compared using Fisher’s LSD test when the Fprob from ANOVA was significant at *P* ≤ 0.05. Correlations of FFD between years also used Genstat. The *G*-test was used to test whether the cytotype composition of progeny of open-pollinated mother plants deviated significantly from that expected from the potential parent plants. Genome size variation within cytotypes from Britain and Ireland was analysed by ANOVA and *t*-test.

## Results

### Cytotypes—DNA content, geographic distribution and genome size variation

#### DNA content.

Nuclear 2C DNA contents confirmed a polyploid series with 2*n* = 2, 4, 5 and 6x cytotypes (Table 1) with mean values of 2.40, 4.51, 5.56, and 6.53 pg for 2*n* = 34, 68, 85 and 102 chromosomes, respectively. The 1Cx value of the monoploid genome declined with increasing ploidy. The data for fresh samples (Table 1) and all samples (Fig. 3) both show a range of DNA content within a cytotype, which was confirmed by chromosome counts for all euploids except diploids. The ranges for fresh samples were slightly narrower than those for the full sample set, and equivalent to about half of a Cx value. Three groups of aneuploids were detected, 4–5x, 5–6x and potential 6–7x.

#### Geographic distribution.

The distribution of cytotyped individuals in Britain and Ireland (Fig. 4A) shows many new locations, and confirms that hexaploids have a predominantly western and tetraploids have an eastern distribution, that tetraploids do not occur in Ireland and that diploids are absent. Previous occasional observations of hexaploids outside their normal western range, sympatric with tetraploids at Wanlockhead/Leadhills (WLC), Teesdale (TC) and Cheddar Gorge (CHE) were confirmed and the two former found to be much more extensive than previously determined. Two new large clusters of sympatric hexaploids were found in the Pennines: one north (close to Alston—AC), and one south (centred on Wensleydale—WC) of the known Teesdale cluster. Only 13 pentaploids were found in this study of >1700 individuals, 11 of these occurred in the mixed population contact zones at WLC, TC, CHE, AC and WC which all have long histories of mineral extraction and contain unusual plant species (e.g. Houston *et al.* 2009; Vallejo-Marin 2012; Gater 2018). The remaining two were in tetraploid populations. All of the aneuploids detected were at or adjacent to contact zones. In terms of climate, hexaploids occupied locations in Britain and Ireland with CCI 2–8, whereas tetraploids occurred in Britain at locations with CCI 3–11 (Figs 2B and 4A).

On a broader scale (Fig. 4B), diploids have an easterly distribution, overlapping with tetraploids. Hexaploids were found in northern Spain, Canada and the USA.

#### Within cytotype genome size variation.

Tetraploids from Britain showed no significant regional variation in 1Cx values, but there was a significant difference between populations which were sympatric or allopatric with hexaploids (*t*-test *P* < 0.001). 1Cx values of sympatric tetraploids (1.14 pg) were 1.7 % higher than those of allopatric tetraploids (1.12 pg). The sympatric hexaploids at mine sites in the Pennines and in Scotland also had significantly higher 1Cx values (1.11 pg) (1.9 %) (*P* < 0.001) than their allopatric populations (1.09 pg).

### Chloroplast haplotype variation and distribution

**Figure 6.**
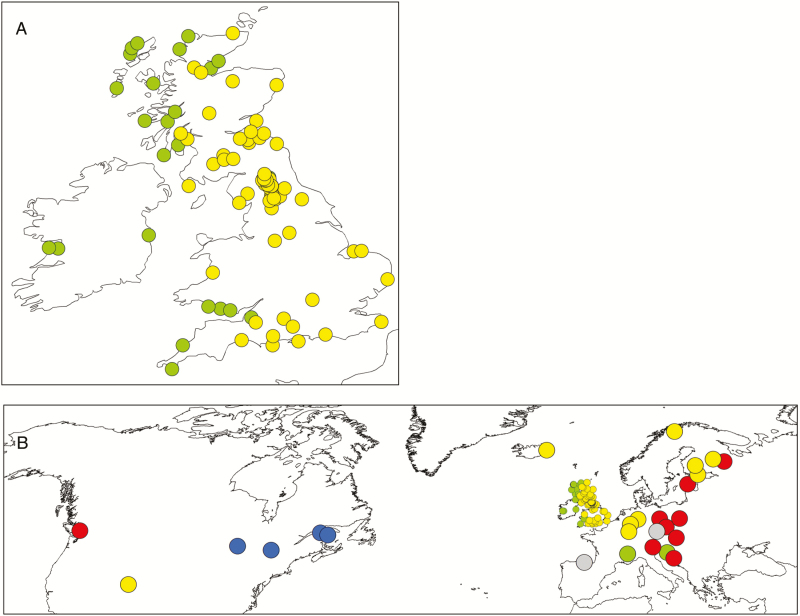
Geographical location of chloroplast DNA haplotype groupings of *C. rotundifolia* (A) in Britain and Ireland and (B) the northern hemisphere. Note that in (B), some eastern European locations have been shifted to avoid overlay of symbols. The colour coding in Fig. 5B provides the key to this figure. See also **Supporting Information—Tables S2****and****S3**.

Combining the three chloroplast loci resulted in 29 chloroplast haplotypes from 126 populations within which there was cytotype and geographic partitioning (Figs 5A and B; 6A and B) **[see****Supporting Information—Tables S2****and****S3****]**. Four groups (labelled A–D) were evident within the haplotype network (Fig. 5A and B). Diploids were exclusively in Group A, and were from mainland Europe (Croatia, Czech Republic and Finland), together with tetraploids from Estonia and Canada. Group B comprised tetraploids from Britain, mainland Europe (Germany and Finland) and Iceland, together with pentaploids and hexaploids from the Pennine clusters, and hexaploids from the Wanlockhead/Leadhills cluster. A single hexaploid from the USA (Colorado) was also placed in this group. Group C was predominantly of hexaploids from Ireland and western Britain (including Cheddar Gorge) with one tetraploid from each of the Czech Republic, France and Teesdale. Intermediate between Group A and Groups B and C is Group D, which contains tetraploids and hexaploids from North America. Two intermediate samples from Spain (hexaploid) (haplotype 20) and the Czech Republic (tetraploid) (haplotype 13) were unresolved between Groups B and C.

### Common garden growth and phenology

#### Survival and growth.

Most of the clones were extremely vigorous and performed far better than in their native habitat. After 1 year, the mean shoot dry weight of tetraploid clones was 7.7 g (range 0.6–19.8 g), with a mean of 29 (range 5–64) flowering stems per plant, and the mean shoot dry weight of hexaploid clones was 6.1 g (range 0.2–21.0 g) with a mean of 27 (range 0–71) flowering stems per plant. The mean shoot dry weight of the single pentaploid clone was 14.8 g, and it was exceptionally floriferous, with an average of 81 flowering stems. Four years after planting, there was 93, 100 and 97 % survival of 4x, 5x and 6x cytotypes, respectively.

#### Flowering phenology.

The 2010 season was cooler than 2009 and fewer growing degree days had accumulated by the start of flowering **[see****Supporting Information—Fig. S2****]**. However, the overall mean FFD of clones and cytotypes was similar in both years (Table 2). The earliest clone in 2009 (northern England 54.3°N—tetraploid) flowered on Day 184 and the earliest in 2010 (south-west England 50.6°N—hexaploid) flowered on Day 185. The latest FFD was more variable between years, corresponding to Day 217 in 2009 and 224 in 2010. In both seasons, the last clones to flower were hexaploids from Bull Island (Republic of Ireland). During 2009, 645.5 and 956.6 GDD had accumulated by the time the first and last clones came into flower, while in 2010, only 569.9 and 929.4 GDD accumulated. The assessment on Day 215 in 2009 showed that most of the early flowering clones were still producing new flowers and flower buds when the late flowering clones came into bloom **[see****Supporting Information—Fig. S1****]** and all clones which had a FFD on or before Day 209 (except one hexaploid from SW England) had swollen seed heads, indicating successful seed set. Most clones continued to flower until late October.

**Table 2.** Mean FFD (day number) of clones in the common garden study in 2009 and 2010, by cytotype and CCI at site of origin. Values within a row are significantly different (by ANOVA^a^ using mean LSD) when they are succeeded by different letters.

	Cytotype									
	Tetraploid		Pentaploid		Hexaploid		All cytotypes		*F* prob	
2009	196.4		197.4		197.8		197.0		0.155	
2010	196.9a		196.8a		199.3b		197.8		<0.010	
										
	Conrad index									
FFD	2	4	5	6	7	8	9	10	11	*F* prob
2009 All	200.7d	196.7bc	200.9d	199.7cd	194.6ab	192.7a	195.3ab	195.0ab	197.6bcd	<0.001
2010 All	202.3ef	199.3cde	203.4ef	199.9de	194.0ab	192.3a	195.7abc	197.2bcd	205.5f	< 0.001
∆ All^b^	1.6abc	2.4c	2.4c	1.3abc	−0.8a	−0.5ab	0.5ab	2.2bc	6.4d	< 0.001
2009 Tet	–	200.2d	196.7bcd	199.7cd	196.3bc	191.8a	195.3ab	195.0ab	197.6bcd	<0.001
2010 Tet	–	203.4de	197.6bc	199.9cd	195.5b	191.6a	195.7b	197.2bd	205.4e	<0.001
∆ Tet^b^	–	3.3c	1.0abc	1.3abc	−0.9a	−0.2ab	0.5abc	2.2bc	6.3d	<0.003
N. Tet	0	2	7	14	6	8	12	4	2	
2009 Hex	200.7b	196.1a	204.5b	–	192.9a	193.2a	–	–	–	<0.001
2010 Hex	202.3c	198.5b	208.5c	–	192.4a	192.4a	–	–	–	<0.001
∆ Hex^b^	1.6ab	2.2b	3.7b	–	−0.7a	−0.7a	–	–	–	<0.001
N. Hex	7	10	8	0	6	6	0	0	0	

^a^One-way ANOVA with unbalanced treatment structure.

^b^Difference in FFD between 2010 and 2009; negative values indicate plants flowering earlier in 2010 than 2009, positive values indicate later flowering in 2010.

#### Cytotype and flowering phenology.

All cytotypes showed a positive correlation between FFD in 2009 and 2010 (*P* < 0.001), and 64 % of the clones varied by <3 days between the 2 years. There was no significant difference between cytotype in their mean FFD in 2009, but in the cooler 2010, hexaploid FFD was slightly but significantly later than the other cytotypes (*P* < 0.001) (Table 2).

#### Flowering phenology and CCI.

In both years, clones from locations with intermediate CCI (CCI 7–10 in 2009 and 7–9 in 2010) flowered significantly earlier than those from other locations (Table 2) **[see****Supporting Information—Fig. S3****]**, and were less delayed in 2010. The CCI at the common garden site is 6. Plants from CCI 2 and 4 were either entirely or predominantly hexaploid and from the Scottish islands or west coast mainland, whereas those from CCI 6, 9, 10 and 11 were entirely tetraploid. The later flowering clones from CCI 10 and 11 were tetraploids from central and south-east England. The late-flowering hexaploids in CCI 5 **[see****Supporting Information—Fig. S3****]** were from Ireland.

### Contact zone progeny

**Figure 7.**
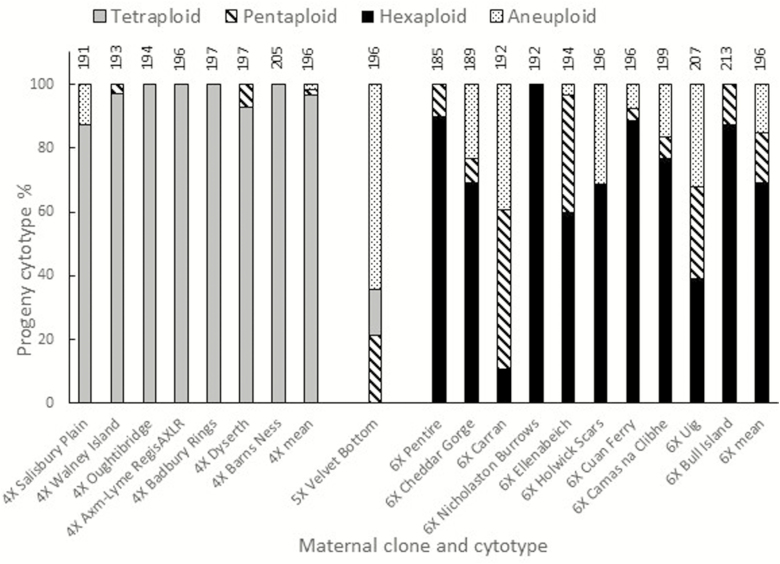
Cytotypes of seedling progeny of tetraploid (4X), pentaploid (5X) and hexaploid (6X) open-pollinated mother plants grown in the common garden, ordered by FFD (shown above the progeny bar) within each maternal cytotype.

The proportion of seedling progeny having the same cytotype as their mother plant varied within and between mother plant cytotypes (Fig. 7), indicating ploidy-dependent differences in cross-compatibility. Progeny of tetraploid mother plants were mostly tetraploid (97 %), whereas those of hexaploid mother plants were more mixed, with mean 69 % hexaploids. Few aneuploids or pentaploids were detected among the progeny of tetraploids, but they comprised 31 % of hexaploid progeny. The single pentaploid mother plant clone was vigorous and fertile and yielded 21 % pentaploid progeny, with the remainder being aneuploids (64 %) and tetraploids (15 %).

All the hexaploid clones in this study were of hexaploid-type Group C chloroplast haplotypes except the Holwick Scars hexaploid from the Teesdale contact zone with tetraploid-type Group B haplotypes (Fig. 5). Despite occurring in a contact zone, this clone was not reproductively isolated from other cytotypes (Fig. 7). The progeny of the hexaploid clones varied considerably in their cytotype proportions and degree of interploid reproductive isolation: the clone from Nicholaston Burrows in Wales yielded 100 % hexaploid progeny, while the clone from Carran in Ireland produced only 10 % hexaploid progeny. These two clones shared the same FFD (Fig. 7), suggesting that the difference in proportion of their progeny is due to maternal characteristics rather than differences in availability of pollen. The FFD of most of the other mother plants also overlapped very closely, and as this species has a long flowering season, the availability of pollen to mother plants would have been very similar.

The proportion of open flowers of the different cytotypes over the flowering season was estimated to be 32 tetraploid:1 pentaploid:14 hexaploid **[see****Supporting Information—Fig. S1****]** and the observed and expected proportions of progeny of the maternal cytoype arising from freely outcrossing mother plants of tetraploid and hexaploid clones **[see****Supporting Information—Table S4****]** showed that both cytotypes had a greater tendency to produce progeny of the same cytotype as themselves than would be expected from random mating (*G*-test) (*P* < 0.001).

## Discussion

This study confirms the predominantly allopatric distribution of hexaploid and tetraploid cytotypes in Great Britain and Ireland. Irish populations were entirely hexaploid. Extensive new populations of sympatric hexaploids were identified in the Pennine Hills. At sympatric locations, pentaploids and aneuploids were found. The allopatric hexaploids of western Britain share cpDNA haplotypes with those in Ireland, but are distinct from British tetraploids, whereas sympatric hexaploids share haplotypes with tetraploids.

The common garden study indicated genotype × environment effects on FFD, related to climate at the home site, but there were no climatic barriers to the growth of ‘western’ hexaploids, and the cytotypes were not phenologically isolated. When cytotypes were brought together at the contact zone, maternal tetraploids produced mostly tetraploid progeny, whereas hexaploids of both ‘western’ and ‘tetraploid’ haplotypes produced many aneuploids and pentaploids.

Integrating these different elements enables us to understand the ancient and contemporary forces shaping the polyploid distribution and the factors affecting the stability of present-day populations.

### Cytogeography and genome size variation

In continental Europe, the distribution of cytotypes is structured. Diploids only occurred in the east (Fig. 4A and B) (supporting previous observations by Böcher 1960; Gadella 1964; Laane *et al.* 1983), while tetraploids had a much wider range, and both occurred in Siberia. Hexaploids were only recorded at the extreme north-western fringes of Europe, in Ireland and parts of Britain, and in northern Spain. Both tetraploids and hexaploids occurred in North America.

Although the distribution in the British Isles is strongly allopatric, large populations of hexaploids occur outside their normal range in proximity to tetraploids at Cheddar Gorge, the Pennines and at Wanlockhead/Leadhills, demonstrating that the two cytotypes occasionally co-exist in nature (Fig. 4A). McAllister (1972) reported hexaploids in the Pennines (at Teesdale) and at Wanlockhead, so these populations are not transient. Most (11 out of 13) of the pentaploids and all the aneuploids occurred where hexaploids and tetraploids are sympatric, indicating that the cytotypes are not completely reproductively isolated at these locations, supporting the results of the contact zone study.

The Cx values of different cytotypes (Table 1) show genome downsizing (Leitch and Bennett 2004) of 5.9 % between diploid and tetraploid, and 9.1 % between diploid and hexaploid. Our data show broad ranges of 2C and 1Cx (monoploid genome) DNA values for each cytotype (Fig. 3). The mean diploid 2C value (2.40 ± 0.11 pg) (Table 1) is higher than that of Slovenian diploids (2.19 pg) (Temsch *et al.* 2010), and the mean 2C value for the (predominantly British) tetraploid (4.51 ± 0.10 pg) (Table 1) is less than that from the Czech Republic (4.66 ± 0.004 pg) (Münzbergová 2009). Bai *et al.* (2012) report a ‘2C’ value (not verified by chromosome counts) of 6.4 pg from the USA, which is similar to our hexaploid values. Such differences may represent genome size variation at regional and continental scales (Lysák *et al.* 2000; Duchoslav *et al.* 2013).

Although there was no regional variation in genome size in British tetraploids, sympatric populations of British tetraploids and hexaploids at mine sites in the Pennines and in Scotland had significantly higher 1Cx values than allopatric populations, but the differences (*c.* 2 %) are small compared with regional variation in *A. oleraceum* of 2.3–18.3 % (Duchoslav *et al.* 2013), though similar to differences (1.6 %) in *Sesleria albicans* (Lysák *et al.* 2000).

### Phylogeography

The segregation of cytotype variation and geographic region within the cpDNA groups (Figs 5 and 6) provides indications of the origins, patterns of migration and evolution of the hexaploid and tetraploid cytotypes in Britain and Ireland. The formation of this genetic structure is likely to have been initiated during the retreat of the British–Irish ice sheet after the last glacial maximum (LGM).

The presence of *C. rotundifolia* seeds in sedimentary records of the cold-stage flora in the late Devensian period from eastern Britain (West 2000) shows that the species was established in Britain while ice sheets were still extensive (Clark *et al.* 2012). *Campanula rotundifolia* has been grown from seed collected from wind-blown debris on St Mary’s glacier, Colorado (Bonde 1969), and debris-covered glaciers may have served as Pleistocene refugia (Fickert *et al.* 2007). If this perennial herbaceous species gained a foothold, and temperatures were sufficiently warm for occasional flowering, its seeds could have been blown long distances across icy surfaces and germinated when conditions became favourable. Glacial fringes could accumulate blown debris from adjacent tundra and provide invasion routes as the climate warmed. The subspecies, *giesekiana*, currently grows, but rarely flowers, north of the Arctic Circle on Svalbard at 78°7′N (Alsos *et al.* 2003), and tetraploid *C. rotundifolia* flowers at 3000 m in the alpine tundra of Colorado (Bingham and Ranker 2000).

At cpDNA loci, the molecular distinctiveness of the western British and Irish hexaploids from British tetraploids (Fig. 5A and B) **[see****Supporting Information—Tables S2****and****S3****]** suggests that they did not originate from British tetraploids. If they had done so (Fig. 1, route c), cpDNA haplotypes would be commonly shared among tetraploid and western hexaploid populations, which is not the case. The western hexaploids probably originated at another location in continental Europe, either from polyploidization of triploids resulting from crossing of diploid and tetraploid in a diploid–tetraploid contact zone (Fig. 1, route b), or from combination of reduced and unreduced tetraploid gametes (route c). It is noteworthy that the single hexaploid found in mainland Europe (Spain) (haplotype 20) is intermediate between the main tetraploid and hexaploid Groups B and C (Fig. 5A), and also that tetraploids from France and Croatia are present in Group C, with the western hexaploids (Figs 5 and 6) **[see****Supporting Information—Tables S2****and****S3****]**. This suggests that European tetraploids are ancestral to the western British and Irish hexaploids, but also that their source populations in continental Europe had undergone a period of isolation prior to recolonization of Britain and Ireland after the LGM. This concurs with findings by Sutherland and Galloway (2018b) that two distinct refugial populations were present in continental Europe but contrasts with their suggestion of a single colonization route from continental Europe through Britain to Ireland.The cpDNA similarity of the hexaploids in Ireland, the Scottish islands and western mainland Britain implies connectivity of populations in these island locations at the time of colonization, i.e. before they were separated by sea-level rise *c.* 15–16 000 years BP (Brooks *et al.* 2011a, b; Clark *et al.* 2012). Hexaploid colonization could have followed similar migration routes to those suggested for Irish oaks (*Quercus robur* and *Q. petraea*) (Kelleher *et al.* 2004) and the ‘Lusitanian’ flora of Ireland (Beatty and Provan 2013, 2014; Beatty *et al.* 2015; Santiso *et al.* 2016), moving from south-western refugia in mainland Europe or on the exposed continental shelf, across the dry Celtic Sea to south-western Britain and southern Ireland, gradually progressing north as conditions warmed and finally colonizing the north-western Scottish mainland and islands between 19 000 and 15 000 years BP—a period of 4000 years for hexaploids to cover a distance of 1000–1800 km, depending upon the location of the original refugia. This is equivalent to 250–450 m year^−1^, comparable to the suggested rates of movement of various tree species during the Holocene of 50–700 m year^−1^ (Birks 1989).

The overall distribution of tetraploids, and their present-day absence from Ireland and most Scottish islands indicates that they followed a more easterly postglacial invasion route, and that their movement to Ireland was probably blocked by sea-level rise. The hexaploid population at Cheddar Gorge (Fig. 4A) may be a remnant of once more extensive ‘western-type’ hexaploid populations. Its haplotype is unique **[see****Supporting Information—Table S3****]** but falls within the Group C western haplotypes (Fig. 5). This south-western British population is within a gorge, surrounded by tetraploids on higher land, and adjacent to the Somerset Levels, a lowland boggy area of inhospitable habitat for this species. Many of the western hexaploids are isolated today by inhospitable terrain—mountain, bog, wetland, intensive agriculture. While the westerly migration route for hexaploids from continental Europe would have closed 15–16 000 years BP, the window of opportunity for further colonization of Britain by tetraploids from continental Europe would have remained open through the North Sea and Channel land bridges until *c.* 10 000 years BP (Brooks *et al.* 2011a, b).

The only hexaploids exhibiting ‘tetraploid-type’ (Group B) chloroplast DNA are from the Pennines (AC, TC, WC) and Leadhills/Wanlockhead (WLC) (Fig. 5A and B) **[see****Supporting Information—Tables S2****and****S3****]**, sympatric with tetraploids and with higher 1Cx values than allopatric hexaploids. A pentaploid from TC was also of Group B haplotype. These data suggest that in certain locations hexaploids are arising from local tetraploids, through combination of reduced and unreduced gametes (Fig. 1, route c), and are not relics of previously more widespread ‘western’ hexaploid populations or the result of human-mediated seed introduction of the western cytotype.

The higher Cx values of sympatric hexaploids with ‘tetraploid-type’ cpDNA might be indicative of their evolutionary pathways differing from western hexaploids, but the tetraploids at these sites also had higher Cx values, suggesting a common cause. Perhaps environmental stresses at these cold metal-contaminated mine sites result in the production of secondary metabolites which interfere with DNA fluorochromes during flow cytometry (Loureiro *et al.* 2006).

### Phenology

Genotype × environment interactions are evident in FFD and response to temperature. First flowering date in the common garden was related to climate (CCI) at a clone’s origin (Fig. 2B and Table 2). Clones from locations with CCI’s similar to that of the common garden flowered earlier than those of more oceanic or continental origin. The most continental tetraploid cytotypes showed delayed flowering in the cooler year 2010 suggesting that they required a greater heat sum than clones from intermediate zones **[see****Supporting Information—Figs S2****and****S3****]**. However, although there is divergence in FFD, this species has a long flowering season, offering opportunities for cross pollination between different cytotypes and populations, and there is no evidence that the cytotypes are phenologically isolated in Britain. While early flowering of plants in Ireland was noted by Jeffree (1960), we found that Irish hexaploids were the last to flower in the common garden (Table 2, CCI 5, hexaploids), which may indicate particularly strong genotype × environment interactions for these eight clones.

### Mechanisms for maintenance of allopatry and sympatry

Although Sutherland and Galloway (2016) found that crosses between tetraploid and hexaploid cytotypes yielded almost entirely pentaploid offspring with either maternal cytotype, our open-pollinated field observations found that tetraploid mothers produced mostly tetraploid progeny, while hexaploid mothers were more variable, producing 14–100 % hexaploid progeny with many pentaploids and aneuploids (Fig. 7). Their study used controlled crosses, while our field-grown plants will have received pollen from multiple donors of the same and different cytotypes and factors such as stigma receptivity, pollen tube growth rates and competition will be influential in determining the cytotype identity when there are multiple donors. The progeny of the pentaploid in our study (from Velvet Bottom, adjacent to Cheddar Gorge) were mostly aneuploid, with 21 % pentaploid and 15 % tetraploid. Although none of our pentaploid progeny were backcrossed to their parental cytotypes, these data indicate that hexaploids will be progressively eliminated in mixed populations, unless new hexaploids constantly arise from mutations of tetraploids. This is supported by McAllister’s (1972) observations that backcrossing of pentaploids (85 chromosomes) produced aneuploids which were very variable in pollen production, and in which at meiosis ~34 chromosome pairs separated regularly while the remaining univalents did not.

Genome imbalance in the seed endosperm (deviation from the ratio of 2 maternal (m):1 paternal (p) genome) is implicated in determining the success of crosses between cytotypes (Ramsey and Schemske 1998; Sonnleitner *et al.* 2013; Sutherland and Galloway 2016). Our open-pollinated study showed that both tetraploid and hexaploid mother plants preferentially produced offspring of the same cytoype as themselves **[see****Supporting Information—Table S4****]**, favouring the maintenance of a 2m:1p ratio. However, maternal hexaploids produced more pentaploid progeny (3m:1p ratio) than did maternal tetraploids (4m:3p), but there was considerable variation between hexaploid mother plants in the outcome.

#### Allopatry.

The common garden study indicates that there are few contemporary climatic barriers to a more easterly distribution of the western hexaploids, although not necessarily across the full range of climate in Britain. The reciprocal study for tetraploids, planting in a hexaploid zone, was not done, but the current distribution of tetraploids in all CCI zones except CCI 2 indicates that the cytotype has a wide climatic range. Thus, climate and phenology do not appear to be significant factors in the maintenance of the allopatry of the tetraploids and western hexaploids.

Furthermore, the progeny data from the contact zone open-pollinated crosses (Fig. 7) show that while overall there is a strong effect of maternal cytotype on offspring cytotype, there are one-sided reproductive barriers, which are in favour of persistence of the tetraploid and to the detriment of the hexaploid. While our range-wide survey of cytotypes found that most pentaploids and all aneuploids were located at or close to contact zones, the contact zone study showed that all but one of the test hexaploids, whether sympatric or allopatric in origin, would hybridize if brought into contact with other cytotypes, and many of these resulting hybrids were aneuploids.

The absence of strong reproductive barriers in hexaploids and lack of significant climatic or phenological differentiation suggests that the spatial separation evident in their current distribution in Britain and Ireland is essential to the persistence of the hexaploid. While many of these hexaploids are protected by their island locations, some occur on mainland Britain, often in locations where the species is less widespread and appears to be in decline (compare Fig. 2A and 4A).

#### Sympatry.

The locations of sympatric populations in the Pennines and at Wanlockhead/Leadhills (where hexaploids have tetraploid-type cpDNA) are coincident with upland locations with relatively harsh local climates and long histories of mining and metal contamination. Metal contamination and stress may increase the production of unreduced gametes (Ramsey and Schemske 1998; Birks and Willis 2008), leading to constant renewal of opportunities for new polyploids, this and the perennial nature of the species may allow persistence of the minority hexaploid cytotype at these sites. Although hexaploids have been present at these sites for >40 years (McAllister 1972), our observations of their progeny at the common garden contact zone show that they have not escaped minority cytotype exclusion. The haplotype of the Cheddar population suggests that it is a remnant of western populations; it also freely crossed at the simulated contact zone. Its survival, like other western populations, may depend upon its physical isolation within the Gorge, whereas other western populations are isolated by habitat or geographic distance.

## Conclusions

Western allopatric hexaploids in Britain and Ireland are genetically distinct from British tetraploids. Their current distribution reflects separate postglacial invasion pathways in the late Devensian period before land masses were isolated by sea-level rise, rather than contemporary climatic limits. Separate invasion pathways are consistent with evidence from other elements of the Irish flora. Some hexaploids are sympatric with tetraploids in upland locations in Britain and share cpDNA haplotypes with them, suggesting that they are of more recent origin. Simulated contact zone studies showed one-sided reproductive barriers, which are in favour of persistence of the tetraploid and to the detriment of the hexaploid. The over-riding factor in maintaining this distinctive allopatric distribution is the geographic isolation of the western hexaploids, which are not phenologically isolated and mostly not resistant to crossing with tetraploids. What drives the local evolution of the new hexaploids remains unclear, but a common feature of the sites where they are present is an upland location and a history of mining and metal contamination. Both historical processes and ongoing evolution continue to shape genetic structure in this globally widespread plant species.

## Supporting Information

The following additional information is available in the online version of this article—

Appendix S1. Supporting Information—methods and collectors.

**Table S1.** Locations of all sampled populations of *Campanula rotundifolia*, and tests conducted.

**Table S2.** Variable nucleotide sites in the chloroplast regions trnH-trnK and trnC-ycf6, and trnD-trnT score.

**Table S3.** Distribution of chloroplast DNA haplotypes by location and cytotype.

**Table S4.** Potential outcomes of crosses between maternal and paternal tetraploid, pentaploid and hexaploid cytotypes.

**Table S5.** Accession and IPEN numbers for *Campanula rotundifolia* seeds supplied by Botanic Gardens.

**Figure S1.** Numbers of flower buds, open flowers and swollen seed heads per plant of each clone on Day 215 in 2009 in relation to the mean first flowering date of the clone in that year.

**Figure S2.** Cumulative growing degree days > 5 °C (broken line) and daily mean temperature (°C) (solid line) in (a) 2009 and (b) 2010 in relation to first flowering dates (ordinal day number) in the common garden study.

**Figure S3.** Relationship between first flowering date (FFD) (ordinal day number) and Conrad’s continentality index of individual tetraploid, hexaploid and pentaploid clones in the common garden in (a) 2009 and (b) 2010, and (c) difference in FFD between 2009 and 2010.

plaa011_suppl_Supplementary_Material

## Data Archiving

Sequence data have been deposited at GenBank: accession numbers KU246054–KU246111**[see****Supporting Information—Table S2****]**. All other data (latitude and longitude location of all cytotypes, common garden data—FFD of each plant and destructive and non-destructive harvest data) have been archived at the Centre for Ecology & Hydrology Environmental Information Data Centre and can be accessed at https://doi.org/10.5285/673b2b31-562c-4604-8776-4a92988867b8.

## Sources of Funding

This work was supported by Natural Environment Research Council (NERC) via the Centre for Ecology & Hydrology through project NEC04202 and the Botanical Society of Britain and Ireland to J.W.; NERC also supported the studentship of J.R.S. The Consejo Nacional de Ciencia y Tecnología (CONACYT) (grant number 314569) supported M.D.-C.

## Contributions by the Authors

J.W. and C.E.J. planned the research, and collected field samples with much help from those listed in **Supporting Information—Appendix S1**. J.W. set up the common garden study, J.R.S. performed the chromosome counts. Flow cytometry was done by J.W., A.P., and M.D.-C. A.P. and S.C. led the molecular lab work, analysed the cpDNA data and contributed to the discussion, analysis and interpretation of the data as a whole. JW wrote the manuscript with input from all other authors.
