# Supplementary material for: Invasion, isolation and evolution shape population genetic structure in Campanula rotundifolia
Source: AoB Plants. 2020 Mar 12;12(2):plaa011. doi: 10.1093/aobpla/plaa011 (PMC7141102; doi:10.1093/aobpla/plaa011)
Supplement: plaa011_suppl_Supplementary_Material [file plaa011_suppl_supplementary_material.pdf]

Supplementary Table S1 Locations of all sampled populations of *Campanula rotundifolia*, and tests conducted. The first number in the cytotype column is the cytotype determined by flow cytometry, (using the genome size limits in Fig. 3) followed by the number of samples tested if > 1, * indicates samples for which chromosomes were counted (Shepherd, 2007). Cytotypes 2, 4, 5, 6, are diploid, tetraploid, pentaploid, and hexaploid. Aneuploids are 4 - 5, 5 - 6 and 6 - 7. In the cpDNA, common garden and common garden progeny columns, the values are the number of samples or clones tested in these studies. Clusters represent areas of several km^2 in the Pennines (Alston AC, Teesdale TC, Wensleydale WC) and at Wanlockhead/Leadhills (WLC) where hexaploid groups occur in proximity to tetraploids and pentaploids, and intensive sampling was done. Data for other locations is merged where latitude and longitude differ by <0.01°. For mapped distribution of these locations see Fig. 4.

Country	Location	Latitude	Longitude	Cytotype (n)	Cp DNA	Common Garden	CG Progeny
Croatia	Boljara Spring, Nature Park Zumberak-Samoborsko Gorge	45.75	15.43	2 (2)	1		
Croatia	Mreznica Canyon, near Primislje	45.18	15.48	2 (4)	1		
Czech Republic	Břehy	50.05	15.58	2	1		
Czech Republic	Dvůr Králové	50.45	15.82	2	1		
Czech Republic	Hořovice	49.83	13.90	2	1		
Czech Republic	Zdechovice	50.01	15.46	2 (2)	1		
Finland	Mekrijärvi Research Station	62.78	30.97	2 (6)*	1		
Russian Federation	Khanty-Mansisysk	60.98	69.01	2 (6)			
Canada	Oldman River[1]	49.92	-111.83	4			
Canada	Olivine	48.93	-66.12	4	1		
Canada	West Vancouver	49.35	-123.17	4	1		
Croatia	Dobra, near Ogulin	45.26	15.23	4	1		
Czech Republic	Klínek	49.80	14.00	4	1		
England	Allendale	54.87	-2.24	4 (6)			
England	Allendale	54.83	-2.23	4 (3)	1		
England	Allenheads	54.81	-2.23	4 (19)	1		
England	Allenheads	54.81	-2.25	4 (2)			
England	Allenheads	54.80	-2.23	4			
England	Alston cluster AC	54.80	-2.41	4 (45)	2		
England	Arkendale	54.22	-1.94	4			
England	Arnside Knott	54.19	-2.84	4 (5)	1		
England	Arreton, Isle of Wight	50.69	-1.24	4	1		
England	Aston Rowant	51.66	-0.95	4			
England	Axmouth-Lyme Regis Undercliffs	50.72	-3.05	4	1	1	1
England	Badbury Rings	50.82	-2.05	4 (9)*	1	2	1
England	Bampton	54.56	-2.74	4 (3)			
England	Bardsea and Birkrigg Common	54.16	-3.10	4 (4)*		2	
England	Barnard Castle	54.54	-1.92	4			
England	Beacon Hill	51.06	-1.42	4	1		
England	Beeley	53.21	-1.59	4 (2)			
England	Beeley hilltop	53.21	-1.58	4			
England	Bellerby	54.33	-1.86	4			
England	Bembridge and Culver Down, Isle of Wight	50.67	-1.11	4 (2)			
England	Bicester	51.86	-0.55	4			
England	Bradley Cross	51.28	-2.76	4 (3)	1		

Country	Location	Latitude	Longitude	Cytotype (n)	Cp DNA	Common Garden	CG Progeny
England	Brampton	54.93	-2.59	4			
England	Brean Down	51.33	-3.02	4*		1	
England	Broad Downs	51.17	0.97	4		1	
England	Burnhope	54.74	-2.25	4			
England	Burnhope	54.74	-2.23	4			
England	Burrington Combe	51.32	-2.75	4 (4)			
England	Burton Leonard	54.06	-1.51	4			
England	Buxton	53.24	-1.97	4 (2)			
England	Calva Hill	54.40	-2.00	4 (4)			
England	Celeron	54.61	-2.76	4			
England	Chapman's Pool	50.59	-2.06	4 (8)*	1	2	
England	Chillerton Down	50.65	-1.33	4 (2)			
England	Chollerford	55.02	-2.09	4*			
England	Cissbury Rings	50.86	-0.38	4	1		
England	Coalcleugh	54.79	-2.32	4			
England	Devil's Dyke	50.89	-0.20	4			
England	Ditchling Beacon	50.90	-0.11	4			
England	Dolebury Warren	51.33	-2.79	4 (2)			
England	Dovedale	53.06	-1.78	4 (4)			
England	Dufton	54.62	-2.49	4 (3)			
England	Dufton	54.63	-2.48	4			
England	Dukerdale	54.44	-2.29	4			
England	Dunnerholme	54.21	-3.21	4 (3)*	1	1	
England	Dunwich Heath	52.26	1.62	4 (2)	1		
England	East Fremington	54.38	-1.90	4			
England	Easterside Lane, nr Hawnby	54.30	-1.16	4*	1	1	
England	Easton Royal	51.34	-1.71	4*		1	
England	Eggleston	54.60	-2.01	4			
England	Eglingham	55.44	-1.85	4			
England	Eskmeals Dunes	54.34	-3.41	4			
England	Ewebank Scar	54.46	-2.34	4			
England	Ewebank Scar	54.49	-2.25	4			
England	Formby	53.54	-3.09	4 (6)			
England	Foulden Common	52.57	0.60	4			
England	Foulstone Road	53.41	-1.67	4			
England	Fremington	54.37	-1.88	4	1		
England	Freshwater Bay, Isle of Wight	50.67	-1.50	4			
England	Gallows Hill	51.85	-0.60	4			
England	Gamblesby	54.76	-2.58	4 (3)			
England	Gamblesby	54.74	-2.58	4 (2)			
England	Garrigill	54.73	-2.22	4			
England	Garrigill south	54.74	-2.36	4			
England	Garsdale	54.31	-2.44	4			
England	Gilsland	55.00	-2.69	4			
England	Gilsland	55.00	-2.67	4			
England	Grattondale	53.15	-1.69	4 (4)			
England	Great Dunfell	54.64	-2.49	4 (7)			
England	Greenhead and Gilsland	54.98	-2.55	4 (2)			

Country	Location	Latitude	Longitude	Cytotype (n)	Cp DNA	Common Garden	CG Progeny
England	Ham Hill	50.95	-2.74	4			
England	Hawnby	54.31	-1.14	4 (2)		2	
England	Heathcote	53.15	-1.78	4			
England	Heights of Abraham	53.12	-1.56	4 (2)			
England	Helbeck	54.54	-2.32	4			
England	High Bradfield	53.44	-1.62	4		1	
England	High Skears Farm	54.64	-2.08	4			
England	High Sunderland	55.57	-2.84	4			
England	Hindon	54.62	-1.92	4 (3)			
England	Hoggarths	54.41	-2.20	4			
England	Holkham	52.98	0.78	4 (2)*	1	2	
England	Holme Dunes	52.97	0.53	4 (4)*	1	1	
England	Holwick Scar	54.64	-2.13	4 (4)*			
England	Honister Road	54.55	-3.25	4			
England	Honister Road	54.56	-3.22	4			
England	Honister Road	54.52	-3.17	4 (3)			
England	Honister Road	54.52	-3.21	4 (3)			
England	Howden Reservoir	53.43	-1.77	4 (3)			
England	Hullo Bridge	54.27	-1.82	4			
England	Hungry Hall	54.63	-2.13	4			
England	Ingleton	54.58	-1.73	4	1		
England	Ivinghoe Beacon	51.84	-0.61	4			
England	Keisley	54.60	-2.44	4 (3)			
England	Keld	54.40	-2.17	4			
England	Killhope	54.78	-2.30	4			
England	Killhope Wheel	54.78	-2.26	4			
England	Kirkland	54.68	-2.55	4 (4)			
England	Kirkstone	54.44	-2.95	4 (2)	1		
England	Knock Fell	54.68	-2.44	4 (2)			
England	Lakenheath	52.37	0.63	4 (3)*		1	
England	Langdon Hills	51.55	0.42	4			
England	Langthwaite	54.42	-1.99	4 (3)			
England	Lindisfarne Island	55.68	-1.82	4	1		
England	Lonkley Bank	54.89	-2.26	4 (2)			
England	Macclesfield	53.24	-2.03	4 (5)*	1	3	
England	Malham	54.07	-2.15	4 (2)*	1	2	
England	Manystones Lane	53.09	-1.64	4			
England	Melmerby	54.73	-2.59	4			
England	Middleton in Teesdale	54.63	-2.07	4			
England	Millerdale	53.09	-1.79	4 (3)			
England	Minninglow	53.12	-1.71	4 (5)			
England	Minninglow	53.12	-1.69	4			
England	Mungrisford	54.68	-2.98	4*		1	
England	Murton	54.59	-2.42	4 (11)			
England	Murton	54.60	-2.41	4 (3)			
England	New Alston near Haydon Bridge	54.99	-2.24	4			
England	Ninebanks	54.86	-2.33	4 (2)			
England	Old Vicarage Farm	54.38	-1.86	4			

Country	Location	Latitude	Longitude	Cytotype (n)	Cp DNA	Common Garden	CG Progeny
England	Oughtibridge	53.44	-1.56	4	1	1	1
England	Ousby	54.70	-2.60	4 (4)			
England	Ousby	54.71	-2.57	4			
England	Penrith Cemetery	54.67	-2.75	4			
England	Pulpit Hill	51.74	-0.80	4	1		
England	Revlin	54.64	-2.12	4			
England	Richmond	54.42	-1.75	4 (2)			
England	Richmond	54.40	-1.73	4			
England	Rievaulx	54.26	-1.11	4 (3)*		2	
England	River West Allen	54.88	-2.34	4 (4)			
England	Rookhope	54.80	-2.18	4			
England	Rookhope Trail	54.78	-2.09	4 (2)			
England	Rookhope Village	54.78	-2.10	4			
England	Salisbury Plain	51.26	-1.71	4 (3)	1	1	1
England	Scout Scar	54.30	-2.79	4 (4)			
England	Shipham	51.30	-2.78	4			
England	Silecroft	54.22	-3.36	4*		1	
England	Sinderhope	54.86	-2.24	4 (5)	1		
England	Sinderhope	54.84	-2.24	4 (3)			
England	Skirwith	54.69	-2.56	4 (2)			
England	Slit Wood	54.75	-2.15	4 (2)	1		
England	Snaiza Gill on Gayles Moor	54.45	-1.87	4			
England	South Foreland Cliffs, Deal	51.18	1.41	4	1		
England	St Bees Head	54.49	-3.61	4 (2)*			
England	St Catherine's Hill	51.05	-1.31	4			
England	Stanhope - Rookhope	54.75	-2.05	4			
England	Steel Rigg Crag[1]	55.00	-2.39	4 (4)*			
England	Stockbridge Down	51.11	-1.47	4			
England	Stublick Corner & Langley Chimney	54.94	-2.25	4 (2)			
England	Teesdale cluster TC	54.64	-2.21	4 (23)	5		
England	Twyford Down	51.03	-1.28	4			
England	Upper Eden Valley	54.50	-2.23	4			
England	Upper Weardale	54.74	-2.18	4			
England	Upper Weardale	54.74	-2.10	4			
England	Velvet Bottom	51.29	-2.71	4 (2)			
England	Wadsworth	53.74	-1.98	4			
England	Walney Island	54.12	-3.27	4 (3)*		1	1
England	Watendlath	54.56	-3.13	4			
England	Wavering Down	51.30	-2.84	4*		1	
England	Wensleydale Cluster WC	54.29	-2.05	4 (4)	1		
England	West Stonesdale	54.41	-2.18	4 (2)			
England	Winder Fell	54.33	-2.54	4			
England	Windy Hill, Eggleston	54.61	-2.01	4			
England	Wold Fell	54.26	-2.32	4			
Estonia	Himmiste, Lümanda, Saaremaa	58.27	21.98	4	1		
Finland	Enontekion Lappi, Kilpisjarvie, Saana2	69.04	20.86	4*	1		
Finland	Lammi	61.03	25.02	4 (4)	1		
Finland	Mekrijärvi roadside	62.78	30.97	4 (3)	1		

Country	Location	Latitude	Longitude	Cytotype (n)	Cp DNA	Common Garden	CG Progeny
Finland	Suomenlinna	60.14	24.99	4 (2)*	1		
France	Les deux Alpes[1]	45.00	6.12	4	1		
Germany	Altenahr[2]	50.52	7.00	4	1		
Germany	Marsberg Westheim Dahlberg[2]	51.46	8.85	4	1		
Germany	Stillenbergskopf, Warstein, Munster[2]	49.88	11.64	4			
Germany	Traubenberg[2]	50.56	6.60	4	1		
Iceland	Múlasýsla, Geitagerði[2]	65.10	-14.78	4	1		
Russian Federation	Altay[2]	50.50	87.60	4			
Russian Federation	Kondinsky-Ozera	60.87	63.49	4			
Scotland	Abbey St Bathans	55.85	-2.33	4			
Scotland	Aberlady	56.04	-2.87	4 (9)*	1	2	
Scotland	Abington	55.48	-3.71	4 (5)			
Scotland	Abriachan	57.39	-4.42	4 (2)			
Scotland	Abriachan	57.41	-4.41	4 (2)			
Scotland	Abriachan	57.43	-4.38	4			
Scotland	Arisaig	56.94	-5.85	4			
Scotland	Armadale roadside	58.55	-4.07	4			
Scotland	Arthur's Seat	55.95	-3.15	4 (7)*			
Scotland	Ashie Moor	57.36	-4.33	4			
Scotland	Auchengillan	56.00	-4.38	4 (5)			
Scotland	Aviemore	57.18	-3.83	4 (4)			
Scotland	Aviemore	57.20	-3.83	4			
Scotland	Ayr	54.44	-4.66	4			
Scotland	Balmedie Beach	57.25	-2.04	4 (2)	1	1	
Scotland	Ballantrae	55.09	-5.01	4			
Scotland	Ballantrae	55.15	-4.98	4			
Scotland	Ballater	57.05	-3.11	4 (2)			
Scotland	Ballater	57.04	-3.05	4			
Scotland	Ballochdowan	55.06	-5.00	4			
Scotland	Balmoral	57.04	-3.22	4			
Scotland	Balmoral	57.07	-3.22	4			
Scotland	Barns Ness	55.99	-2.44	4 (8)*	1	2	1
Scotland	Bellendean	55.43	-3.00	4			
Scotland	Ben Lawers	56.53	-4.25	4 (9)			
Scotland	Ben Lawers	56.52	-4.26	4 (6)	1		
Scotland	Bettyhill roadside	58.52	-4.23	4			
Scotland	Black Bridge	57.69	-4.72	4			
Scotland	Blackburn	55.19	-2.85	4 (3)			
Scotland	Blackburn	55.19	-2.83	4 (2)			
Scotland	Boarhills[1]	56.32	-2.70	4 (4)*	1		
Scotland	Bogallan Wood	57.52	-4.27	4			
Scotland	Braemar	57.00	-3.31	4			
Scotland	Braes of Foss	56.67	-4.01	4			
Scotland	Braes of Foss	56.64	-4.01	4			
Scotland	Brockhouse Fort and Fountainhall	55.75	-2.93	4 (18)*	1		
Scotland	Brora Dunes	58.01	-3.84	4 (2)			
Scotland	Buckie	57.69	-2.93	4			
Scotland	Bught Hill	55.38	-3.30	4			

Country	Location	Latitude	Longitude	Cytotype (n)	Cp DNA	Common Garden	CG Progeny
Scotland	by Garve	57.61	-4.68	4			
Scotland	Cairngorm	57.06	-4.11	4 (2)			
Scotland	Carham Station	55.62	-2.34	4			
Scotland	Carrbridge	57.28	-3.81	4			
Scotland	Castle Moffat	55.91	-2.64	4*			
Scotland	Castlefairn	55.16	-4.00	4			
Scotland	Castlelaw	55.86	-3.23	4 (4)*	1		
Scotland	Catlodge	56.99	-4.25	4 (2)			
Scotland	Catlodge	56.97	-4.24	4			
Scotland	Catlodge	56.95	-4.24	4			
Scotland	Chanonry Point	57.57	-4.10	4			
Scotland	Chesters Hill Fort	55.99	-2.79	4 (2)			
Scotland	Clach Ard, Isle of Skye	57.58	-6.36	4			
Scotland	Cladich	56.35	-5.08	4			
Scotland	Clatteringshaws	55.05	-4.28	4* (2)			
Scotland	Coire Garb	57.03	-3.86	4			
Scotland	Collydene	55.74	-2.90	4 (3)			
Scotland	Comrie	57.57	-4.66	4			
Scotland	Contin, by graveyard	57.56	-4.59	4			
Scotland	Corrievuic	57.51	-5.01	4			
Scotland	Corsewall	55.02	-5.15	4 (2)			
Scotland	Corsewall	54.99	-5.12	4			
Scotland	Corsewall	54.99	-5.14	4			
Scotland	Cowal Peninsula	55.83	-5.21	4*	1	1	
Scotland	Craigdarroch	55.20	-4.09	4*	1		
Scotland	Croft Crunie Farm Tore	57.54	-4.33	4			
Scotland	Cromdale	57.35	-3.52	4	1	1	
Scotland	Cruden Bay beach	57.41	-1.85	4 (2)			
Scotland	Cullicudden2	57.63	-4.31	4			
Scotland	Cullicudden3	57.64	-4.28	4			
Scotland	Dalmary	56.12	-4.39	4			
Scotland	Dawyck	55.60	-3.35	4			
Scotland	Dere Street	55.41	-2.38	4 (5)			
Scotland	Dere Street	55.39	-2.37	4 (3)			
Scotland	Dere Street	55.42	-2.37	4 (2)			
Scotland	Dunagoil	55.73	-5.04	4 (8)			
Scotland	Dunnet Bay	58.60	-3.35	4 (3)			
Scotland	Dunning	56.21	-3.60	4			
Scotland	Dunning	56.24	-3.59	4(3)			
Scotland	Duthil	57.30	-3.74	4			
Scotland	E Loch Glascarnoch	57.69	-4.79	4			
Scotland	East Loch Mullardoch	57.70	-4.79	4			
Scotland	East Lomond	56.24	-3.28	4			
Scotland	East Lomond	56.24	-3.26	4			
Scotland	Elgin	57.67	-3.16	4			
Scotland	Elgin	57.68	-3.12	4			
Scotland	Enterkin Burn, Lowther hills	55.33	-3.78	4			
Scotland	Enterkin Pass	55.44	-3.35	4			

Country	Location	Latitude	Longitude	Cytotype (n)	Cp DNA	Common Garden	CG Progeny
Scotland	Ervie	54.97	-5.14	4			
Scotland	Ervie	54.89	-5.15	4			
Scotland	Essich	57.44	-4.24	4 (2)			
Scotland	Essich	57.42	-4.26	4 (2)			
Scotland	Essich	57.41	-4.27	4			
Scotland	Ettrick Valley	55.48	-3.01	4			
Scotland	Fairburn	57.54	-4.55	4			
Scotland	Fife Ness	56.28	-2.59	4			
Scotland	Findhorn	57.66	-3.62	4			
Scotland	Findlatter Castle	57.69	-2.77	4 (2)			
Scotland	Findonmains	57.62	-4.33	4			
Scotland	Firbush Point	56.48	-4.27	4			
Scotland	Fochabers	57.64	-3.11	4			
Scotland	Fochabers	57.65	-3.06	4			
Scotland	Forvie	57.34	-1.95	4 (2)		1	
Scotland	Foveran Dunes	57.31	-2.00	4 (3)			
Scotland	Foyers	57.25	-4.49	4			
Scotland	Foyers	57.22	-4.50	4 (2)			
Scotland	Gairlochy	56.87	-5.06	4			
Scotland	Gairlochy	56.91	-5.00	4			
Scotland	Galashiels	55.61	-2.82	4*			
Scotland	Garve	57.61	-4.68	4			
Scotland	Garbat	57.69	-4.71	4			
Scotland	Glen Douglas	56.15	-4.72	4			
Scotland	Glen Etive	56.63	-4.89	4			
Scotland	Glen Feshie	57.01	-3.90	4 (21)			
Scotland	Glen Roy	56.91	-4.81	4 (2)			
Scotland	Glen Roy	56.89	-4.84	4			
Scotland	Glenlivet	57.35	-3.30	4			
Scotland	Glenlivet	57.36	-3.27	4			
Scotland	Glenlivet	57.37	-3.25	4			
Scotland	Glenlivet	57.42	-3.16	4			
Scotland	Glenmuick	57.01	-3.07	4			
Scotland	Glenmuick	56.98	-3.12	4			
Scotland	Glen Turret	56.40	-3.88	4 (2)			
Scotland	Glen Turret	56.42	-3.91	4 (2)			
Scotland	Glenquithle near Pennan	57.67	-2.26	4			
Scotland	Glenshee	56.84	-3.44	4			
Scotland	Hartfell	55.52	-3.06	4			
Scotland	Hartsgarth Burn	55.22	-2.82	4 (2)			
Scotland	Heights of Inchvannie	57.61	-4.52	4			
Scotland	Heights of Keppoch	57.60	-4.51	4			
Scotland	Hermitage	55.25	-2.78	4			
Scotland	Hilton of Delnies	57.59	-3.94	4			
Scotland	Holmhead	55.15	-4.04	4			
Scotland	Innerleithen	55.68	-3.05	4			
Scotland	Insh	57.10	-3.95	4			
Scotland	Insh Marshes	57.08	-4.02	4			

Country	Location	Latitude	Longitude	Cytotype (n)	Cp DNA	Common Garden	CG Progeny
Scotland	Inver	58.51	-4.22	4 (6)			
Scotland	Inver	58.52	-4.24	4 (5)			
Scotland	Inverarnan	56.33	-4.72	4 (4)			
Scotland	Inverchoran	57.51	-4.90	4			
Scotland	Inverfarigaig	57.30	-4.40	4 (2)			
Scotland	Inverfarigaig	57.29	-4.42	4			
Scotland	Irvine	55.60	-4.68	4			
Scotland	Kemnay	57.18	-2.42	4		1	
Scotland	Kershope burn	55.14	-2.78	4			
Scotland	Kilham	55.58	-2.19	4(3)			
Scotland	Kilminning Nature Reserve	56.27	-2.60	4			
Scotland	Kirk Yetholm	55.54	-2.26	4 (4)			
Scotland	Kirkhouse	55.55	-3.09	4			
Scotland	Kirkland	55.20	-3.85	4*			
Scotland	Kirkudbright	54.83	-4.03	4*		1	
Scotland	Knowehead	55.19	-4.17	4*		1	
Scotland	Laggan	57.02	-4.30	4			
Scotland	Laggan	57.01	-4.35	4			
Scotland	Laggan	57.00	-4.36	4			
Scotland	Laggan	57.01	-4.37	4			
Scotland	Laggan	57.01	-4.41	4			
Scotland	Laggan	57.03	-4.48	4			
Scotland	Laggan	57.03	-4.53	4			
Scotland	Laggan	57.03	-4.50	4			
Scotland	Laggan	57.02	-4.44	4			
Scotland	Lagnalean	57.44	-4.30	4			
Scotland	Lang Burn	55.33	-2.74	4			
Scotland	Langholm Hills	55.18	-2.94	4 (2)			
Scotland	Lassintulloch Forest	56.69	-4.12	4			
Scotland	Lassintulloch Forest	56.69	-4.10	4			
Scotland	Lassintulloch Forest	56.68	-4.05	4			
Scotland	Learmouth	57.13	-2.58	4			
Scotland	Links of Greenland	58.60	-3.35	4 (4)			
Scotland	Little Assynt	58.17	-5.15	4			
Scotland	Little Garve	57.62	-4.70	4			
Scotland	Loch Achilty	57.57	-4.64	4			
Scotland	Loch Ashie	57.39	-4.28	4			
Scotland	Loch Ashie	57.37	-4.30	4			
Scotland	Loch Beannacharain	57.51	-4.97	4			
Scotland	Loch Doon	55.30	-4.39	4			
Scotland	Loch Doon	55.28	-4.40	4			
Scotland	Loch Doon	55.26	-4.39	4			
Scotland	Loch Doon	55.23	-4.38	4 (2)			
Scotland	Loch Doon	55.22	-4.40	4			
Scotland	Loch Glascarnoch	57.71	-4.83	4	1		
Scotland	Loch Glascarnoch	57.72	-4.89	4			
Scotland	Loch Leathan, Isle of Skye	57.49	-6.17	4			
Scotland	Loch Lomond	56.06	-4.52	4			

Country	Location	Latitude	Longitude	Cytotype (n)	Cp DNA	Common Garden	CG Progeny
Scotland	Loch nan Eun	58.16	-5.22	4 (5)			
Scotland	Loch Rannoch	56.71	-4.07	4			
Scotland	Loch Rannoch	56.69	-4.43	4			
Scotland	Loch Rannoch	56.68	-4.47	4			
Scotland	Loch Rannoch	56.68	-4.51	4			
Scotland	Loch Rannoch	56.69	-4.53	4			
Scotland	Loch Rannoch	56.68	-4.44	4			
Scotland	Loch Rannoch	56.68	-4.41	4			
Scotland	Loch Rannoch	56.68	-4.34	4			
Scotland	Loch Rannoch	56.71	-4.10	4 (2)			
Scotland	Loch Rannoch	56.69	-4.25	4			
Scotland	Loch Rannoch	56.69	-4.16	4			
Scotland	Loch Rannoch	56.70	-4.13	4			
Scotland	Loch Rannoch	56.70	-4.16	4			
Scotland	Loch Rannoch	56.70	-4.26	4			
Scotland	Loch Rannoch	56.69	-4.32	4			
Scotland	Loch Rannoch	56.69	-4.35	4			
Scotland	Loch Rannoch	56.69	-4.37	4			
Scotland	Loch Venachar	56.23	-4.28	4			
Scotland	Lochmaben	55.13	-3.44	4			
Scotland	Lossiemouth	57.72	-3.34	4			
Scotland	Lumphanen	57.12	-2.70	4			
Scotland	Macduff	57.67	-2.47	4 (2)			
Scotland	Maidens	55.33	-4.83	4			
Scotland	Mark of Luce	54.90	-4.68	4*			
Scotland	Melvich	58.56	-3.92	4 (2)			
Scotland	Mochrum	54.83	-4.64	4*			
Scotland	Monkton	55.51	-4.62	4			
Scotland	Mulchaich	57.58	-4.38	4			
Scotland	Mull of Galloway	54.64	-4.88	4 (4)*	1	2	
Scotland	Nairn	57.59	-3.82	4			
Scotland	New Aberdour	57.66	-2.20	4		1	
Scotland	Newtonmore	57.07	-4.14	4 (2)			
Scotland	Newtonmore	57.07	-4.12	4			
Scotland	Newtonmore	57.07	-4.14	4			
Scotland	Ormidale	55.99	-5.20	4 (2)*	1	1	
Scotland	Over Bohespic	56.72	-4.06	4			
Scotland	Parr Beach	58.53	-4.21	4			
Scotland	Portsoy	57.67	-2.61	4			
Scotland	Powmill	56.17	-3.56	4 (3)			
Scotland	Prestwick Beach	55.51	-4.62	4			
Scotland	Raddery	57.60	-4.16	4			
Scotland	Ralia	57.04	-4.15	4			
Scotland	Rankle Burn	55.43	-3.07	4			
Scotland	Rattray Head	57.61	-1.83	4			
Scotland	Rattray Road	57.61	-1.86	4			
Scotland	Reay	58.57	-3.79	4 (2)			
Scotland	Reay 2019	58.56	-3.76	4			

Country	Location	Latitude	Longitude	Cytotype (n)	Cp DNA	Common Garden	CG Progeny
Scotland	Rosehaugh	57.57	-4.21	4			
Scotland	Roseisle Forest	57.67	-3.50	4			
Scotland	Rosemarkie	57.59	-4.14	4			
Scotland	Ruthven	57.51	-2.81	4 (3)			
Scotland	Scrabster	58.61	-3.55	4 (2)	1		
Scotland	Scalpsie Beach	55.78	-5.10	4 (5)			
Scotland	Selkirk	55.54	-2.82	4			
Scotland	Smailholm Tower	55.60	-2.58	4 (3)			
Scotland	Sourhope	55.47	-2.26	4 (8)			
Scotland	Sourhope	55.49	-2.30	4			
Scotland	South Clunes	57.41	-4.43	4 (2)			
Scotland	South Clunes	57.42	-4.42	4			
Scotland	Spittal	56.74	-3.40	4 (2)			
Scotland	Spittal	56.77	-3.41	4			
Scotland	Spittal	56.92	-3.41	4			
Scotland	Spittal	56.96	-3.41	4			
Scotland	St Abbs	55.89	-2.14	4*			
Scotland	St Fergus cemetery	57.55	-1.81	4 (2)		1	
Scotland	Straiton	55.30	-4.54	4			
Scotland	Straiton	55.27	-4.47	4			
Scotland	Stranraer	54.94	-5.00	4 (3)			
Scotland	Strathconon	57.51	-4.97	4 (3)			
Scotland	Strathconon	57.55	-4.76	4			
Scotland	Strathconon	57.55	-4.84	4			
Scotland	Strathgarve Lodge	57.61	-4.68	4			
Scotland	Strathpeffer	57.61	-4.51	4 (2)			
Scotland	Strathpeffer	57.61	-4.49	4 (2)			
Scotland	Strathblane	55.98	-4.32	4 (3)			
Scotland	Talla Water	55.45	-3.33	4 (2)			
Scotland	Terally bay	54.73	-4.92	4*		1	
Scotland	Thornhill	55.35	-3.73	4			
Scotland	Thurso	58.58	-3.53	4 (2)			
Scotland	Tombrech Farm	56.51	-4.19	4			
Scotland	Tomintoul	57.12	-3.14	4			
Scotland	Tomintoul	57.18	-3.23	4			
Scotland	Tomintoul	57.22	-3.30	4			
Scotland	Tomintoul	57.27	-3.32	4			
Scotland	Torness	57.33	-4.36	4			
Scotland	Torness	57.31	-4.38	4			
Scotland	Torphins	57.10	-2.62	4			
Scotland	Torrachilty by Contin	57.58	-4.59	4	1		
Scotland	Trinafor	56.79	-4.09	4 (4)	1		
Scotland	Trinafour	56.80	-4.09	4 (2)			
Scotland	Trinafour	56.77	-4.08	4 (2)			
Scotland	Trinafour	56.75	-4.09	4			
Scotland	Trislaig	56.82	-5.13	4 (4)			
Scotland	Troon	55.53	-4.65	4			
Scotland	Tullochgrue	57.16	-3.79	4 (7)			

Country	Location	Latitude	Longitude	Cytotype (n)	Cp DNA	Common Garden	CG Progeny
Scotland	Tummel	56.73	-4.09	4			
Scotland	Tummel	56.73	-4.08	4			
Scotland	Tynehead	55.83	-2.97	4 (6)*	1		
Scotland	Wanlockhead/Leadhills cluster WLC	55.41	-3.78	4 (65)	1		
Scotland	West Hatton, by Westhill	57.16	-2.25	4		1	
Scotland	West Lomond Hills	56.24	-3.26	4			
Scotland	White Castle Fort	55.91	-2.62	4 (2)*			
Scotland	Whiteadder Water	55.88	-2.58	4 (2)*	1		
Scotland	Whitebridge	57.20	-4.51	4			
Scotland	Whitehills	57.68	-2.59	4			
Scotland	Whitehills	57.67	-2.57	4			
Scotland	Whitshields Knowe, Langholm	55.16	-3.00	4			
Scotland	Yellowcraig	56.06	-2.77	4			
Spain	Dorria	42.33	2.05	4			
Spain	Dorria	42.32	2.07	4 (3)			
Spain	Masella	42.29	1.86	4			
USA	Chippewa County, WI1	46.45	-84.34	4	1		
USA	Denver	39.74	-104.99	4			
USA	Durango	37.38	-107.76	4			
USA	Florissant	38.92	-105.28	4			
Wales	Aberystwyth	52.43	-4.02	4 (4)*	1	2	
Wales	Bedwelly churchyard	51.70	-3.21	4			
Wales	Bronaber	52.87	-3.89	4 (3)			
Wales	Clydach	51.80	-3.12	4 (2)			
Wales	Dolgellau	52.73	-3.82	4			
Wales	Dyserth	53.28	-3.40	4 (4)*		2	1
Wales	Eglwyseg	53.00	-3.18	4			
Wales	Gwaelod-y-Brithdir	51.70	-3.24	4			
Wales	Gwynfryn	53.07	-3.11	4			
Wales	Hirnant	52.80	-3.40	4			
Wales	Lake Vyrnwy	52.80	-3.58	4 (2)			
Wales	Llanarmon Dyffryn Ceiriog	52.89	-3.27	4			
Wales	LLangynog	52.83	-3.40	4			
Wales	Llanrhaeadr ym Mochant	52.83	-3.28	4			
Wales	Llanrhaeadr ym Mochant	52.84	-3.27	4			
Wales	Llanrhaeadr ym Mochant	52.87	-3.27	4			
Wales	Llanymawddwy	52.79	-3.56	4			
Wales	Minera	53.06	-3.10	4 (4)			
Wales	Minera	53.05	-3.12	4 (2)			
Wales	Minera	53.05	-3.09	4			
Wales	Mynydd Llandygai	53.17	-4.09	4			
Wales	Newborough Warren	53.15	-4.36	4			
Wales	Nicholaston Burrows[1]	51.58	-4.17	4		1	
Wales	Offa's Dyke path	53.04	-3.14	4			
Wales	Pen-y-Pass	53.08	-4.02	4			
England	Rookhope	54.80	-2.18	4 - 5 (3)			
England	Teesdale cluster TC	54.66	-2.22	4 - 5	1		

Country	Location	Latitude	Longitude	Cytotype (n)	Cp DNA	Common Garden	CG Progeny
England	Wensleydale Cluster WC	54.29	-2.05	4 - 5			
England	Ewebank Scar	54.46	-2.34	4 - 5			
England	Alston cluster AC	54.79	-2.39	5 (4)	3		
England	Wensleydale Cluster WC	54.26	-2.21	5			
England	Teesdale cluster TC	54.66	-2.22	5 (4)	3		
England	Velvet Bottom CHE	51.30	-2.71	5		1	1
Scotland	Brockhouse Fort	55.75	-2.93	5			
Scotland	Loch Rannoch	56.68	-4.29	5			
Scotland	Wanlockhead/Leadhills cluster WLC	55.41	-3.78	5			
England	Alston cluster AC	54.79	-2.39	5 - 6			
England	Howgills Cumbria	54.35	-2.56	5 - 6			
England	Rookhope	54.80	-2.21	5 - 6			
England	Wensleydale Cluster WC	54.26	-2.21	5 - 6 (2)	1		
England	Teesdale cluster TC	54.65	-2.25	5 - 6			
Scotland	Wanlockhead/Leadhills cluster WLC	55.41	-3.80	5 - 6 (3)			
Canada	Ottawa	45.70	-76.18	6	1		
Canada	Perce	48.52	-64.23	6	1		
England	Allendale	54.87	-2.24	6			
England	Alston cluster AC	54.79	-2.39	6 (65)	4		
England	Cheddar Gorge	51.29	-2.75	6 (14)*	1	5	1
England	Coalcleugh	54.79	-2.32	6 (3)			
England	Coalcleugh	54.80	-2.31	6			
England	Coalcleugh	54.82	-2.31	6			
England	Keld	54.40	-2.17	6			
England	Lizard	49.99	-5.24	6 (9)	1		
England	Middleton in Teesdale	54.63	-2.07	6			
England	Moorhouse	54.69	-2.38	6			
England	Pentire Head	50.59	-4.92	6 (7)	1	2	1
England	Pryhill Farm	54.80	-2.39	6			
England	Scout Scar	54.30	-2.79	6			
England	Teesdale cluster TC	54.63	-2.22	6 (89)	4	1	1
England	Wensleydale cluster WC	54.29	-2.05	6 (15)	3		
Republic of Ireland	Ballyryan	53.05	-9.36	6 (2)			
Republic of Ireland	Bull Island	53.38	-6.13	6 (3)*	1	3	1
Republic of Ireland	Bundoran	54.48	-8.30	6 (2)			
Republic of Ireland	Carran	53.04	-9.05	6 (6)*	2	3	1
Republic of Ireland	Doolin cliffs	52.99	-9.41	6			
Republic of Ireland	Doolin sandhills	53.01	-9.40	6			
Republic of Ireland	Eagles Rock	53.08	-9.00	6		2	
Republic of Ireland	Glengesh Pass	54.73	-8.48	6			
Republic of Ireland	Kilclooney	54.80	-8.44	6			
Republic of Ireland	Killult	55.13	-8.13	6			
Republic of Ireland	Kiltoorish	54.82	-8.49	6 (5)			
Republic of Ireland	Mullaghmore	54.46	-8.45	6 (2)			
Republic of Ireland	Pollan Bay	55.29	-7.39	6			

Country	Location	Latitude	Longitude	Cytotype (n)	Cp DNA	Common Garden	CG Progeny
Republic of Ireland	Portnoo	54.84	-8.48	6			
Republic of Ireland	Poulsallagh	53.06	-9.35	6	1		
Republic of Ireland	Rossbeg	54.83	-8.50	6 (2)			
Republic of Ireland	Rossbeg	54.81	-8.51	6 (2)			
Republic of Ireland	Rosses Point	54.31	-8.57	6 (3)			
Republic of Ireland	Streedagh	54.40	-8.57	6 (4)			
Northern Ireland	Ballintoy	55.23	-6.33	6			
Northern Ireland	Ballintoy	55.24	-6.35	6 (6)			
Northern Ireland	Ballyvoy	55.21	-6.16	6 (2)			
Northern Ireland	Benome Dunes	55.17	-6.86	6			
Northern Ireland	Benome Dunes	55.17	-6.88	6 (3)			
Northern Ireland	Binevenagh	55.12	-6.91	6			
Northern Ireland	Cushendall	55.05	-6.13	6 (4)			
Northern Ireland	Cushendun	55.16	-6.03	6 (2)			
Northern Ireland	Derrygonnelly	54.40	-7.91	6			
Northern Ireland	Derrygonnelly	54.40	-7.89	6 (6)			
Northern Ireland	Giant's Causeway	55.24	-6.52	6			
Northern Ireland	Glenariff	55.06	-5.98	6 (7)			
Northern Ireland	Glenarm	54.96	-5.92	6 (7)			
Northern Ireland	Magheramorne	54.82	-5.78	6			
Northern Ireland	Rathlin Island	55.28	-6.19	6 (5)			
Northern Ireland	Rathlin Island	55.26	-6.19	6 (20)			
Northern Ireland	Silent Valley	54.13	-6.00	6			
Northern Ireland	Torr	55.20	-6.06	6 (3)			
Scotland	Aonach Mhor	56.84	-4.98	6			
Scotland	Dalmore level crossing	57.99	-4.17	6			
Scotland	Achmelvich Beach	58.17	-5.31	6			
Scotland	Achnaha	56.73	-6.13	6			
Scotland	Achnaha	56.73	-6.15	6 (2)			
Scotland	Achnaha	56.74	-6.16	6			
Scotland	after Shin Falls	57.94	-4.41	6			
Scotland	Alness	57.69	-4.26	6 (3)	1		
Scotland	Ardhalanish Bay, Mull	56.29	-6.22	6			
Scotland	Ardmore	57.84	-4.18	6 (2)			
Scotland	Baligrundle	56.51	-5.52	6 (4)			
Scotland	Balintore	57.75	-3.92	6			
Scotland	Balnacoll	58.07	-4.03	6			
Scotland	Balnacoll	58.07	-4.06	6			
Scotland	Balnagall	57.82	-3.92	6			
Scotland	Balnaha	57.80	-3.89	6			
Scotland	Balnakiel	57.59	-4.76	6 (9)			
Scotland	Barr	55.22	-4.72	6 (2)			
Scotland	Beinn na Drise, Isle of Mull	56.51	-6.10	6 (2)			
Scotland	Ben Nevis	56.81	-5.07	6			
Scotland	Bloody Bay, Isle of Mull	56.64	-6.12	6 (4)			
Scotland	Borremor, Isle of Lewis	57.84	-7.01	6			
Scotland	Brora Ford	58.02	-3.91	6			
Scotland	Brora roadside	58.04	-3.94	6 (2)			

Country	Location	Latitude	Longitude	Cytotype (n)	Cp DNA	Common Garden	CG Progeny
Scotland	Camas na Clibhe, Isle of Lewis	58.22	-6.96	6 (3)*	1	3	1
Scotland	Carsaig, Isle of Mull	56.32	-5.99	6 (3)			
Scotland	Carsaig, Isle of Mull	56.34	-5.99	6 (3)			
Scotland	Clach Toll	58.19	-5.34	6 (5)	1		
Scotland	Clachan	56.54	-5.46	6 (2)			
Scotland	Clachandhu, Isle of Mull	56.44	-6.13	6 (2)			
Scotland	Coul	57.93	-4.00	6 (5)			
Scotland	Crofts of Kingscausway	57.78	-4.07	6			
Scotland	Cromarty	57.67	-4.08	6			
Scotland	Cuan Ferry and Isle of Luing	56.27	-5.64	6 (10)*	1	3	1
Scotland	Cullicudden roadside	57.62	-4.33	6			
Scotland	Culcairn	57.71	-4.23	6			
Scotland	Culrain	57.91	-4.40	6			
Scotland	Culrain	57.93	-4.42	6			
Scotland	Dalmore	58.00	-4.19	6			
Scotland	Dalmore	57.99	-4.18	6			
Scotland	Dingwall	57.63	-4.37	6			
Scotland	Dornoch Beach	57.88	-4.01	6 (6)	1	1	
Scotland	Durness	58.53	-4.79	6			
Scotland	Edderton Hill	57.82	-4.17	6			
Scotland	Ellenabeich	56.30	-5.65	6 (4)*	1	3	1
Scotland	Fidden, Isle of Mull	56.31	-6.37	6 (8)			
Scotland	Fort George	57.58	-4.06	6 (2)			
Scotland	Frackersaig	56.51	-5.53	6			
Scotland	Gleann Seilisdeir, Isle of Mull	56.42	-6.13	6 (2)			
Scotland	Glen Etive	56.63	-4.89	6			
Scotland	Glen Etive	56.62	-4.94	6			
Scotland	Glen Tarbert, Strontian	56.69	-5.43	6 (2)			
Scotland	Glenglass	57.67	-4.34	6			
Scotland	Gometra	56.48	-6.29	6			
Scotland	Gordonbush	58.06	-3.95	6			
Scotland	Grasswards	55.84	-4.83	6 (4)			
Scotland	Great Cumbrae	55.78	-4.93	6 (4)			
Scotland	Great Cumbrae	55.79	-4.93	6			
Scotland	Great Cumbrae	55.79	-4.91	6 (2)			
Scotland	Greenock	55.93	-4.77	6			
Scotland	Haunn Eriskay	57.09	-7.30	6 (2)			
Scotland	Helmsdale	58.13	-3.66	6			
Scotland	Helmsdale	58.13	-3.66	6 (2)			
Scotland	Hopeman	57.71	-3.43	6			
Scotland	Inver	57.82	-3.92	6			
Scotland	Inver	58.52	-4.24	6 (2)			
Scotland	Inver House by Garbisdale	57.94	-4.43	6			
Scotland	Inveran	57.95	-4.43	6 (2)			
Scotland	Iona	56.32	-6.41	6 (3)	1		
Scotland	Isle of Arran, Glean Eason, Biorach	55.69	-5.27	6 (2)	1		
Scotland	Isle of Arran, Glen Catacol, Glen Diomhan	55.67	-5.31	6 (2)			
Scotland	Keoldale Green, Kyle of Durness	58.55	-4.78	6			

Country	Location	Latitude	Longitude	Cytotype (n)	Cp DNA	Common Garden	CG Progeny
Scotland	Kerrera	56.40	-5.52	6 (6)			
Scotland	Kincardine by Ardgay	57.87	-4.36	6			
Scotland	Lairg	58.02	-4.40	6			
Scotland	Lairg	58.01	-4.38	6			
Scotland	Lairg	58.01	-4.40	6			
Scotland	Linsidecraig	57.94	-4.44	6			
Scotland	Loch Hallan, South Uist	57.17	-7.40	6	1		
Scotland	Loch na Clais	58.38	-5.07	6			
Scotland	Macrihanish	55.43	-5.73	6 (3)*	1	3	
Scotland	Moultavie	57.71	-4.30	6			
Scotland	Muie	58.01	-4.27	6			
Scotland	Mullach a' Bhreun-Leitir	57.89	-4.92	6			
Scotland	near Balnacoll	58.07	-3.98	6			
Scotland	Nedd	58.24	-5.12	6			
Scotland	Nonikiln	57.71	-4.25	6			
Scotland	Oakbank, Isle of Mull	56.43	-5.69	6 (3)			
Scotland	Oldshoremore	58.48	-5.08	6			
Scotland	Ormsaig, Isle of Mull	56.34	-6.15	6 (4)			
Scotland	Oykel Bridge	57.97	-4.74	6 (2)			
Scotland	Pennyghael, Isle of Mull	56.36	-6.06	6 (5)			
Scotland	Port Ramsay	56.55	-5.45	6 (2)			
Scotland	Port Uilleum	57.84	-3.82	6			
Scotland	Rogart	58.00	-4.14	6			
Scotland	Rogart	57.99	-4.15	6			
Scotland	Sandwood Bay	58.53	-5.06	6 (2)	1		
Scotland	Sanna Bay	56.75	-6.18	6 (5)			
Scotland	Savary	56.55	-5.86	6 (2)			
Scotland	Scotsburn	57.75	-4.15	6			
Scotland	Shandwick	57.74	-3.93	6			
Scotland	Sligachan, Isle of Skye	57.29	-6.18	6	1		
Scotland	Smoo Cave	58.56	-4.72	6 (3)			
Scotland	Straiton	55.28	-4.51	6			
Scotland	Strath of Brora	58.06	-4.11	6			
Scotland	Strath of Pitcalnie	57.73	-3.98	6			
Scotland	Strath Oykel	57.95	-4.49	6			
Scotland	Strathcarron	57.89	-4.46	6 (2)			
Scotland	Strathcarron	57.89	-4.55	6 (2)			
Scotland	Strathcarron	57.89	-4.37	6			
Scotland	Strathcarron	57.89	-4.44	6			
Scotland	Strathcarron	57.89	-4.42	6			
Scotland	Strathcarron	57.88	-4.42	6			
Scotland	Strathcarron	57.90	-4.51	6			
Scotland	Strathcarron	57.90	-4.49	6			
Scotland	Strathcarron	57.89	-4.47	6			
Scotland	Stronacroibh	56.55	-5.44	6 (3)			
Scotland	Strontian	56.73	-5.54	6 (4)			
Scotland	Tonnel Hill	55.77	-4.92	6 (3)			
Scotland	Traigh na Beirigh, Isle of Lewis	58.22	-6.93	6*	1	1	

Country	Location	Latitude	Longitude	Cytotype (n)	Cp DNA	Common Garden	CG Progeny
Scotland	Traigh Niosaboist, Isle of Lewis	57.86	-6.98	6			
Scotland	Tulloch	57.90	-4.35	6			
Scotland	Uig, Isle of Lewis	58.18	-7.03	6 (3)*	1	3	1
Scotland	Ulva	56.49	-6.21	6			
Scotland	Wanlockhead/Leadhills cluster WLC	55.41	-3.78	6 (60)	2		
Scotland	Wellgreens	57.90	-4.40	6			
Scotland	Wilkhaven	57.85	-3.79	6			
Spain	Potes	43.43	-3.82	6*	1		
USA	Colorado State[1]	39.38	-105.35	6	1		
Wales	Golan	52.96	-4.17	6			
Wales	Gwastadnant	53.09	-4.06	6			
Wales	Kenfig	51.51	-3.74	6	1		
Wales	Nicholaston Burrows[1]	51.58	-4.17	6 (9)*	1	4	1
Wales	Ogmore	51.47	-3.63	6 (2)	1		
Wales	Penmorfa	52.95	-4.18	6			
Wales	Snowdon	53.07	-4.08	6 (4)			
England	Teesdale cluster TC	54.63	-2.22	6 - 7 (7)			
England	Wensleydale cluster WC	54.29	-2.05	6 -7			

[1] Commercial seedlot
[2] Botanic Garden seedlot, further details in **Supplementary Table S5**

Supplementary Table S2 Variable nucleotide sites in the chloroplast regions trnH-trnK and trnC-ycf6, and trnD-trnT score. Genbank accession numbers are given (Acc #) for each chloroplast region for every haplotype. Nucleotide position is indicated vertically and refers to the location of each variable site in each sequence. Dashes (-) indicate deletions. Question marks (?) signify missing data. Sequences are grouped into haplotypes based on sequence similarity (H1-29). Colour coding corresponds to the groups in Fig. 5b and 6. Haplotypes coloured grey correspond to those unresolved between groups B and C, in Fig. 5b.

			Nucleotide position of polymorphic site (base pairs)																																																	
			trn*H*-trn*K*																trn*C*-ycf6																																		
Haplotype	Cytotype		0	0	0	0	0	0	0	0	0	0	0	0	3	4	4		0	0	0	0	0	0	0	0	0	0	0	0	1	2	2	2	2	2	2	2	2	2	2	2	2	3	4	4	4	4	4	5	5	5	trnD-trnT score
		Acc #	0	1	5	5	5	6	6	6	6	6	6	6	9	0	1	Acc #	0	0	1	5	7	8	8	8	9	9	9	9	9	0	1	2	2	2	2	3	3	3	3	3	6	5	1	4	7	7	9	0	8	8	
			1	8	5	8	9	0	1	2	3	4	5	6	7	0	0		1	2	7	3	9	0	1	4	5	6	7	8	9	5	4	6	7	8	9	0	1	2	3	4	4	7	9	4	3	5	0	6	2	5	
H1	6	KU246083	T	T	A	-	-	-	-	-	-	-	-	-	T	C	T	KU246054	G	T	C	T	-	-	-	-	T	G	T	C	G	G	C	T	T	T	G	A	T	A	T	T	T	A	G	A	A	C	C	C	A	G	1
H2	6	KU246084	T	T	A	-	-	-	-	-	-	-	-	-	C	T	T	KU246055	G	T	C	T	-	-	-	-	T	G	T	C	G	G	C	T	T	T	G	A	T	A	T	T	T	A	G	A	A	C	C	C	A	G	1
H3	6	KU246085	T	T	A	-	-	-	-	-	-	-	-	-	C	T	T	KU246056	C	T	C	T	G	G	T	-	T	G	T	C	G	G	C	T	T	T	G	A	T	A	T	T	T	A	G	A	A	C	C	C	A	G	1
H4	6	KU246086	T	T	A	-	-	-	-	-	-	-	-	-	C	T	T	KU246057	G	T	C	T	-	-	-	-	T	G	T	C	A	G	C	T	T	T	G	A	T	A	T	T	T	A	G	A	A	C	C	C	A	G	4
H5	6	KU246087	T	T	A	-	-	-	-	-	-	-	-	-	C	T	T	KU246058	G	T	C	T	-	-	-	-	T	G	T	C	A	G	C	T	T	T	G	A	T	A	T	T	T	A	G	A	A	C	G	C	A	G	4
H6	4	KU246088	T	T	A	-	-	-	-	-	-	-	-	-	C	T	T	KU246059	G	T	C	T	G	G	T	-	T	G	T	C	G	G	C	T	T	T	G	A	T	A	T	T	T	A	G	A	A	C	C	C	A	A	4
H7	4	KU246089	T	T	A	-	-	-	-	-	-	-	-	-	C	T	T	KU246060	G	T	C	T	G	G	T	-	T	G	T	C	G	G	C	T	T	T	G	A	T	A	T	T	T	A	G	A	A	C	C	C	A	G	4
H8	4, 6	KU246090	T	T	A	-	-	-	-	-	-	-	-	-	C	T	T	KU246061	G	T	C	T	G	G	T	-	T	G	T	C	G	G	C	T	T	T	G	A	T	A	T	T	T	A	G	A	A	C	C	C	A	G	1
H9	4	KU246091	G	G	A	-	-	-	-	-	-	-	-	-	C	T	C	KU246062	G	T	C	T	G	G	T	-	T	G	T	C	G	G	C	T	T	T	G	A	T	A	T	T	T	A	G	A	A	C	C	C	A	G	3
H10	4	KU246092	G	G	A	-	-	-	-	-	-	-	-	-	C	T	C	KU246063	G	T	G	T	G	G	T	-	T	G	T	C	G	G	C	T	T	T	G	A	T	A	T	T	T	A	G	A	A	C	C	C	A	G	4
H11	6	KU246093	T	T	A	G	A	T	A	T	T	A	A	T	C	T	T	KU246064	G	T	C	T	-	-	-	-	T	G	T	C	G	G	C	T	T	T	G	A	T	A	T	T	T	A	G	A	A	C	C	C	A	G	1
H12	4	KU246094	T	G	C	-	-	-	-	-	-	-	-	-	C	T	?	KU246065	G	T	C	G	G	G	T	T	-	-	-	-	G	G	C	T	T	T	G	A	T	A	T	T	T	A	G	A	A	C	C	C	A	G	?
H13	4	KU246095	T	G	C	-	-	-	-	-	-	-	-	-	C	T	C	KU246066	G	T	C	T	G	G	T	-	T	G	T	C	G	G	C	T	T	T	G	A	T	A	T	T	T	A	G	A	A	C	C	A	A	G	4
H14	2	KU246096	T	G	C	-	-	-	-	-	-	-	-	-	C	T	C	KU246067	G	T	C	G	G	G	T	-	-	-	-	-	G	G	C	T	T	T	G	A	T	A	T	T	T	T	G	A	A	C	C	C	A	G	1
H15	2	KU246097	T	G	C	-	-	-	-	-	-	-	-	-	C	T	C	KU246068	G	T	C	G	G	G	T	-	-	-	-	-	G	G	C	T	T	T	G	A	T	A	T	T	T	A	G	A	A	C	C	C	A	G	3
H16	4	KU246098	T	G	C	-	-	-	-	-	-	-	-	-	C	T	C	KU246069	G	T	C	G	G	G	T	-	-	-	-	-	G	G	C	T	T	T	G	A	T	A	T	T	T	A	G	A	A	A	C	C	A	G	3
H17	2	KU246099	T	G	C	-	-	-	-	-	-	-	-	-	C	T	C	KU246070	G	T	C	G	G	G	T	-	-	-	-	-	G	G	C	T	T	T	G	A	T	A	T	T	G	A	G	A	A	C	C	C	A	G	2
H18	4, 6	KU246100	T	G	A	-	-	-	-	-	-	-	-	-	C	T	C	KU246071	G	T	C	T	G	G	T	-	-	-	-	-	G	G	C	T	T	T	G	A	T	A	T	T	T	A	G	A	A	C	C	C	-	G	3
H19	4, 6	KU246101	T	G	A	-	-	-	-	-	-	-	-	-	C	T	C	KU246072	G	T	C	T	G	G	T	-	-	-	-	-	G	G	C	T	T	T	G	A	T	A	T	T	T	A	G	A	A	C	C	C	A	G	3
H20	6	KU246102	T	G	A	-	-	-	-	-	-	-	-	-	C	T	C	KU246073	G	T	C	T	G	G	T	-	T	G	T	C	G	G	C	T	T	T	G	A	T	A	T	T	T	A	G	A	A	C	C	C	A	G	1
H21	4	KU246103	G	G	A	-	-	-	-	-	-	-	-	-	C	T	C	KU246074	A	A	C	T	G	G	T	-	T	G	T	C	G	G	C	T	T	T	G	A	T	A	T	T	T	A	G	A	A	C	C	C	A	G	4
H22	4	KU246104	G	G	A	-	-	-	-	-	-	-	-	-	C	T	C	KU246075	G	T	C	T	G	G	T	-	T	G	T	C	G	A	C	T	T	T	G	A	T	A	T	T	T	A	G	A	A	C	C	C	A	G	4

**H23**	**4, 6**	**KU246105**	G	G	A	-	-	-	-	-	-	-	-	-	C	T	C	KU246076	G	T	C	T	G	G	T	-	T	G	T	C	G	A	C	T	T	T	G	A	T	A	T	T	T	A	G	A	A	C	C	C	A	G	3
**H24**	**6**	**KU246106**	G	G	A	-	-	-	-	-	-	-	-	-	C	T	C	KU246077	G	T	C	T	G	G	T	-	T	G	T	C	G	G	C	T	T	T	G	A	T	A	T	T	T	A	C	T	A	C	C	C	A	G	4
**H25**	**4, 5**	**KU246107**	G	G	A	-	-	-	-	-	-	-	-	-	C	T	C	KU246078	G	T	C	T	G	G	T	-	T	G	T	C	G	G	C	T	T	T	G	A	T	A	T	T	T	A	C	A	G	C	C	C	A	G	4
**H26**	**4, 5**	**KU246108**	G	G	A	-	-	-	-	-	-	-	-	-	C	T	C	KU246079	G	T	C	T	G	G	T	-	T	G	T	C	G	G	C	T	T	T	G	A	T	A	T	T	T	A	G	A	A	C	C	C	A	G	1
**H27**	**6**	**KU246109**	G	G	A	-	-	-	-	-	-	-	-	-	C	T	C	KU246080	G	T	C	T	G	G	T	-	T	G	T	C	G	G	A	-	-	-	-	-	-	-	-	-	T	A	G	A	A	C	C	C	A	G	4
**H28**	**4, 5, 6 and aneuploid**	**KU246110**	G	G	A	-	-	-	-	-	-	-	-	-	C	T	C	KU246081	G	T	C	T	G	G	T	-	T	G	T	C	G	G	C	T	T	T	G	A	T	A	T	T	T	A	G	A	A	C	C	C	A	G	4
**H29**	**4**	**KU246111**	G	G	A	-	-	-	-	-	-	-	-	-	C	T	C	KU246082	G	T	C	T	G	T	T	-	T	G	T	C	G	G	C	T	T	T	G	A	T	A	T	T	T	A	G	A	A	C	C	C	A	G	2

Supplementary Table S3 Distribution of chloroplast DNA haplotypes by location and cytotype. Colour coding corresponds to the groups in Fig. 5b and 6. Haplotypes coloured grey correspond to those unresolved between groups B and C,

					Chloroplast DNA haplotype																												
Latitude	Longitude	Location[1]	Sample name[2]	Cytotype	1	2	3	4	5	6	7	8	9	10	11	12	13	14	15	16	17	18	19	20	21	22	23	24	25	26	27	28	29
49.83	13.90	Cz	Hořovice	2														1															
45.26	15.23	Cr	Bolijara Spring	2															1														
45.18	15.48	Cr	Mreznica Canyon	2															1														
50.05	15.58	Cz	Břehy	2															1														
50.45	15.82	Cz	Dvůr Králové	2															1														
50.01	15.46	Cz	Zdechovice	2															1														
62.78	30.97	Fi	Mekrijärvi Research Station	2																	1												
45.26	15.23	Cr	Dobra river banks, Ogulin	4						1																							
54.86	-2.24	En	Sinderhope	4							1																						
45.00	6.12	Fr	Les deux Alpes	4							1																						
54.75	-2.21	En	Upper Weardale	4								1																					
54.30	-1.16	En	Easterside Lane, nr Hawnby	4									1																				
56.32	-2.70	Sco	Boarhills	4									1																				
51.46	8.85	Ge	Marsberg Westheim Dahlberg	4										1																			
49.35	-123.17	Can	Vancouver	4												1																	
49.80	14.00	Cz	Klinek	4													1																
58.27	21.98	Est	Himmiste, Lümanda, Saaremaa	4																1													
48.93	-66.12	Can	Olivine	4																		1											
46.45	-84.34	USA	Chippewa County, WI	4																			1										
50.86	-0.38	En	Cissbury Rings	4																					1								
50.69	-1.24	En	Arreton, Isle of Wight	4																						1							
50.72	-3.05	En	Axmouth-Lyme Regis Undercliffs	4																						1							
50.59	-2.06	En	Chapman's Pool	4																						1							

					Chloroplast DNA haplotype																												
Latitude	Longitude	Location[1]	Sample name[2]	Cytotype	1	2	3	4	5	6	7	8	9	10	11	12	13	14	15	16	17	18	19	20	21	22	23	24	25	26	27	28	29
52.26	1.62	En	Dunwich Heath	4																						1							
54.65	-2.25	En	TC, Cronkley Fell	4																						1							
52.97	0.53	En	Holme Dunes	4																						1							
51.26	-1.71	En	Salisbury Plain	4																						1							
62.78	30.97	Fi	Mekrijärvi roadside	4																						1							
65.10	-14.78	Ice	Múlasýsla, Geitagerði	4																						1							
57.71	-4.83	Sco	Loch Glascarnoch	4																						1							
51.06	-1.42	En	Beacon Hill	4																							1						
50.82	-2.05	En	Badbury Rings	4																							1						
56.52	-4.27	Sco	Ben Lawers	4																							1						
57.35	-3.52	Sco	Cromdale	4																							1						
58.61	-3.55	Sco	Scrabster	4																							1						
52.98	0.78	En	Holkham	4																									1				
54.64	-2.15	En	TC, Holwick Scar	4																										1			
54.81	-2.23	En	Allenheads	4																												1	
51.28	-2.76	En	Bradley Cross	4																												1	
51.18	1.41	En	South Foreland Cliffs, Deal	4																												1	
54.58	-1.73	En	Ingleton	4																												1	
54.44	-2.94	En	Kirkstone Pass	4																												1	
55.72	-2.01	En	Lindisfarne Island	4																												1	
54.30	-2.02	En	WC, Ballowfield	4																												1	
54.40	-2.17	En	WC, Keld	4																												1	
54.41	-2.13	En	WC, Swinnergill	4																												1	
54.69	-2.38	En	Moorhouse	4																												1	
54.80	-2.46	En	AC, Bayles	4																												1	
54.37	-1.88	En	Fremington	4																												1	

					Chloroplast DNA haplotype																												
**Latitude**	**Longitude**	**Location[1]**	**Sample name[2]**	**Cytotype**	**1**	**2**	**3**	**4**	**5**	**6**	**7**	**8**	**9**	**10**	**11**	**12**	**13**	**14**	**15**	**16**	**17**	**18**	**19**	**20**	**21**	**22**	**23**	**24**	**25**	**26**	**27**	**28**	**29**
54.07	-2.15	En	Malham	4																												1	
54.77	-2.38	En	AC, Garrigill	4																												1	
54.58	-2.22	En	WC, Lunedale	4																												1	
51.74	-0.80	En	Pulpit Hill	4																												1	
54.65	-2.18	En	TC, High Force	4																												1	
53.44	-1.56	En	Oughtibridge	4																												1	
69.04	20.86	Fi	Enontekion Lappi, Kilpisjarvie, Saana2	4																												1	
60.14	24.99	Fi	Suomenlinna	4																												1	
50.52	7.00	Ge	Altenahr	4																												1	
50.56	6.60	Ge	Traubenberg	4																												1	
55.83	-2.97	Sco	Tynehead	4																												1	
57.25	-2.04	Sco	Balmedie Beach	4																												1	
56.04	-2.87	Sco	Aberlady	4																												1	
56.79	4.70	Sco	Trinafor	4																												1	
55.75	-2.93	Sco	Brockhouse Fort	4																												1	
55.99	-2.44	Sco	Barns Ness	4																												1	
55.86	-3.23	Sco	Castlelaw	4																												1	
55.20	-4.09	Sco	Craigdarroch	4																												1	
55.43	-3.76	Sco	WLC, Mine 4	4																												1	
55.99	-5.20	Sco	Ormidale	4																												1	
55.83	-5.21	Sco	Point Farm	4																												1	
57.58	-4.59	Sco	Torachillty	4																												1	
55.88	-2.58	Sco	Whitadder	4																												1	
52.43	-4.02	Wa	Aberystwyth	4																												1	
54.21	-3.21	En	Dunnerholme	4																													1
53.24	-2.03	En	Macclesfield	4																													1

					Chloroplast DNA haplotype																												
Latitude	Longitude	Location[1]	Sample name[2]	Cytotype	1	2	3	4	5	6	7	8	9	10	11	12	13	14	15	16	17	18	19	20	21	22	23	24	25	26	27	28	29
61.03	25.02	Fi	Lammi	4																													1
54.64	-4.88	Sco	Mull of Galloway	4																													1
54.65	-2.25	En	TC, Cronkley Fell	4 - 5																												1	
54.77	-2.39	En	AC, Fiddlers Way, Garrigill	5																										1			
54.82	-2.41	En	AC, Alston	5																												1	
54.79	-2.47	En	AC, Leadgate	5																												1	
54.64	-2.16	En	TC, Holwick Scars	5																												1	
54.65	-2.18	En	TC, High Force	5																												1	
54.65	-2.19	En	TC, High Force	5																												1	
54.26	-2.22	En	WC, Cam Fell	5 - 6																												1	
55.43	-5.73	Sco	Macrihanish	6	1																												
51.51	-3.74	Wa	Kenfig	6		1																											
50.59	-4.92	En	Pentire	6			1																										
57.88	-4.01	Sco	Dornoch Beach	6				1																									
58.53	-5.06	Sco	Sandwood Bay	6				1																									
57.69	-4.26	Sco	Alness	6				1																									
55.69	-5.27	Sco	Isle of Arran	6				1																									
57.29	-6.18	Sco	Sligachan, Isle of Skye	6				1																									
58.19	-5.34	Sco	Clachtoll	6				1																									
56.30	-5.65	Sco	Ellenabeich	6				1																									
56.32	-6.41	Sco	Isle of Iona	6				1																									
56.27	-5.64	Sco	Isle of Luing	6					1																								
53.04	-9.05	RoI	Carran	6		1																											
53.04	-9.05	RoI	Carran	6		1																											
53.06	-9.35	RoI	Poulsallagh	6		1																											
58.22	-6.96	Sco	Camas na Clibhe, Isle of Lewis	6		1																											

					Chloroplast DNA haplotype																												
Latitude	Longitude	Location[1]	Sample name[2]	Cytotype	1	2	3	4	5	6	7	8	9	10	11	12	13	14	15	16	17	18	19	20	21	22	23	24	25	26	27	28	29
57.17	-7.40	Sco	Loch Hallan, S Uist	6		1																											
58.22	-6.93	Sco	Traigh na Beirigh, Isle of Lewis	6		1																											
58.18	-7.03	Sco	Uig, Isle of Lewis	6		1																											
51.57	-4.13	Wa	Nicholaston Burrows	6		1																											
51.47	-3.63	Wa	Ogmore	6		1																											
55.24	-6.35	En	Lizard	6								1																					
53.38	-6.13	RoI	Bull Island	6								1																					
51.29	-2.75	En	Cheddar Gorge	6											1																		
48.52	-64.23	Can	Perce	6																		1											
45.70	-76.18	Can	Ottawa	6																			1										
43.43	-3.82	Spain	Potes	6																				1									
54.40	-2.17	En	WC, Keld	6																							1						
54.70	-2.29	En	TC, Harewood	6																								1					
54.65	-2.19	En	TC, High Force	6																											1		
54.82	-2.41	En	AC, Alston	6																												1	
54.78	-2.36	En	AC, Fiddlers Way, Garrigill	6																												1	
54.79	-2.33	En	AC, Nenthead	6																												1	
54.82	-2.31	En	AC, Carrshield	6																												1	
54.64	-2.16	En	TC, Holwick Scar	6																												1	
54.32	-2.08	En	WC, Askrigg	6																												1	
54.64	-2.15	En	TC, Holwick Scar	6																												1	
54.67	-2.28	En	TC, Herdship Fell	6																												1	
55.38	-3.78	Sco	WLC, SW Wanlockhead	6																												1	
55.42	-3.75	Sco	WLC, E. Leadhills	6																												1	
39.38	-105.35	USA	Colorado	6																												1	

[1] Country codes Ca – Canada, Cr – Croatia, Cz – Czech Republic, En – England, Est – Estonia, Fi – Finland, Fr – France, Ge – Germany, Ice – Iceland, RoI - Republic of Ireland, Sco - Scotland, Wa – Wales

[2] Location codes AC – Alston Cluster, TC – Teesdale Cluster, WC – Wensleydale Cluster, WLC – Wanlockhead/Leadhills Cluster

Supplementary Table S4 Potential outcomes of crosses between maternal and paternal tetraploid, pentaploid and hexaploid cytotypes, with applied correction for the availability of different paternal cytotypes (proportion of number of flowers of each cytotype available on day 215), showing the expected and observed proportions of progeny (observed % calculated from data shown in Fig.7)

		**Paternal cytotype and gametes**			**% progeny cytotype expected and observed**				
**Maternal**		**4**	**5**	**6**		**4**	**5**	**6**	**aneuploid**
**cytotype**	**gametes**	**2 * 2**	**1 * 2 1 * 3**	**2 * 3**					
**4**	**2 * 2**	4 * 4x	2 * 4x	4 * 5x					
			2 * 5x						
**Applying floral**		32 * 4 * 4x	2 * 4x	14 * 4 * 5x	**exp**	69.1	30.9	0	0
**frequency**			2 * 5x		**obs**	96.7	1.5	0	1.8
**correction**									
	**1 * 2**	2 * 4x	1 * 4x	2 * 5x					
**5**	**1 * 3**	2 * 5x	2 * 5x	2 * 6x					
			1 * 6x						
**Applying floral**		32 * 2 * 4x	1 * 4x	14 * 2 * 5x					
**frequency**		32 * 2 * 5x	2 * 5x	14 * 2 * 6x	**exp**	34.6	50	15.4	0
**correction**			1 * 6x		**obs**	14.3	21.4	0	64.3
**6**	**2 * 3**	4 * 5x	2 * 5x	4 * 6x					
			2 * 6x						
**Applying floral**		32 * 4 * 5x	2 * 5x	14 * 4 * 6x	**exp**	0	69.1	30.9	0
**frequency**			2 * 6x		**obs**	0	15.6	69.1	15.3
**correction**									

Supplementary Table S5 Accession and IPEN numbers for *Campanula rotundifolia* seeds supplied by Botanic Gardens.

Country	*Location*	*Botanic garden accession no.*	*IPEN number*
Finland	Enontekion Lappi, Kilpisjarvie, Saana	Helsinki Botanic Garden 051/2005	
Germany	Altenahr	University of Bonn Botanic Garden	DE-0-BONN-13952
Germany	Marsberg Westheim Dahlberg	Munster Botanic Garden, no. 237 Index seminum 2009 BG Nr 547	DE-0-MSTR-SA 8851
Germany	Stillenbergskopf, Warstein, Munster	University Bayreuth Botanic Garden 394/09	
Germany	Traubenberg	University of Bonn Botanic Garden	DE-0-BONN-13842
Iceland	Múlasýsla, Geitagerði	Reykjavik Botanic Garden	IS-0-REYK-2008/011
Russian Federation	Altay	New York Botanic Garden 427/2008	

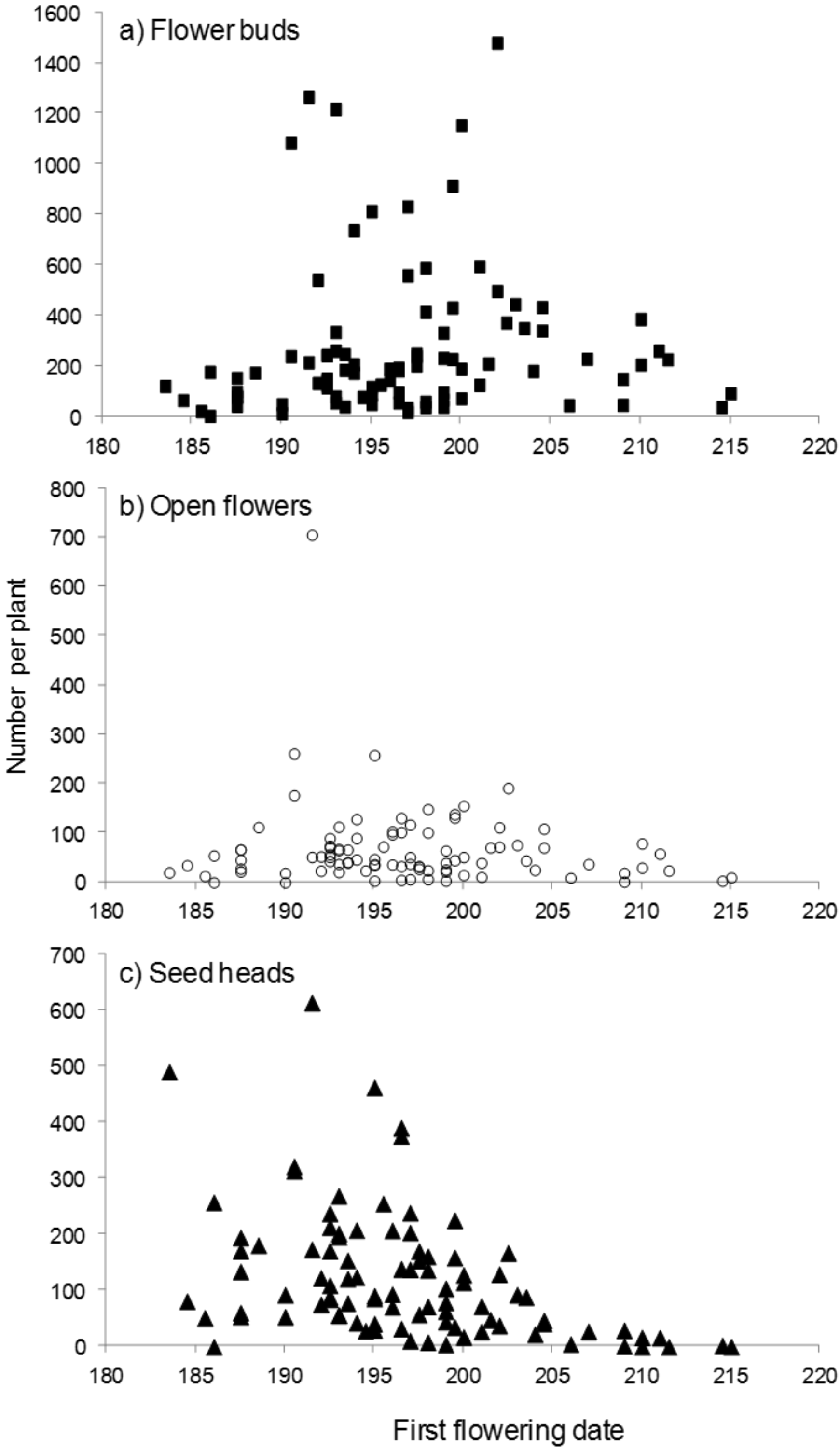

Supplementary Figure S1 Numbers of flower buds, open flowers and swollen seed heads per plant of each clone on day 215 in 2009 in relation to the mean first flowering date of the clone in that year.

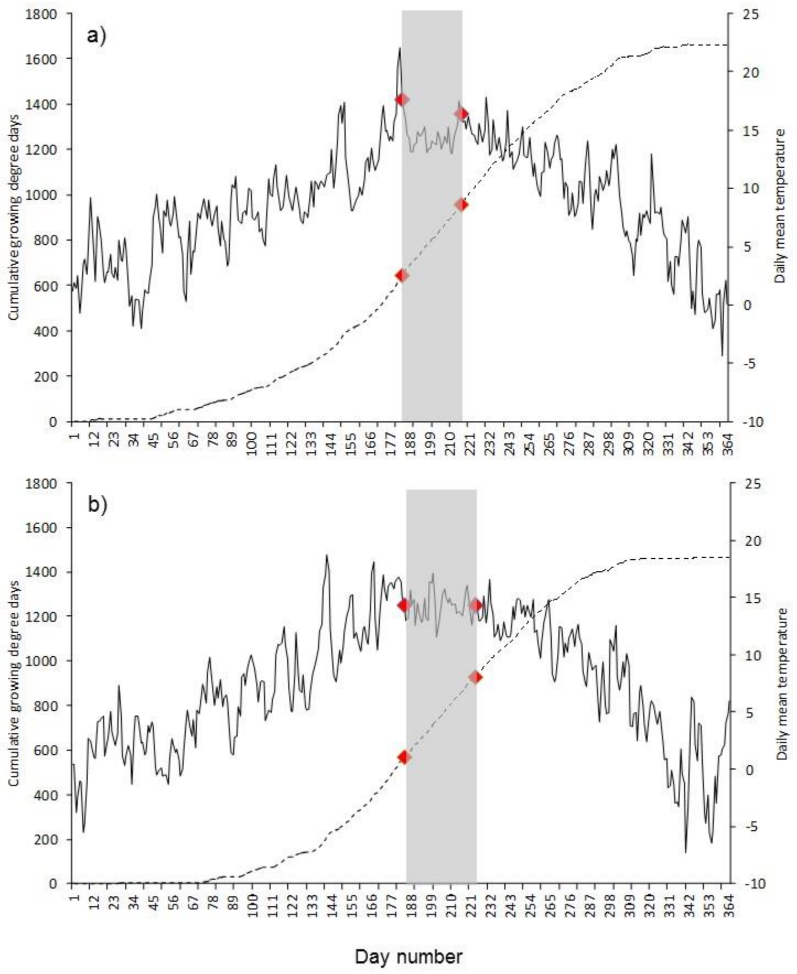

Supplementary Figure S2 Cumulative growing degree days > 5 °C (broken line) and daily mean temperature (°C) (solid line) in (a) 2009 and (b) 2010 in relation to first flowering dates (Ordinal day number) in the common garden study. Grey band indicates the duration of the period during which the first flowering of all clones occurred, also highlighted in red on the degree day and temperature graphs.

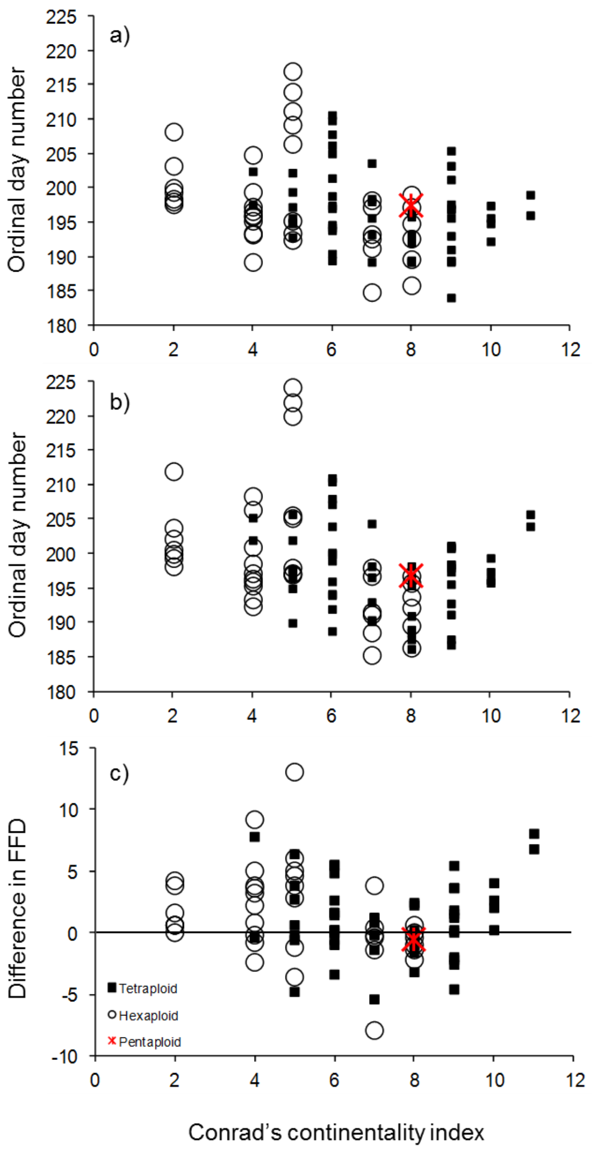

Supplementary Figure S3 Relationship between first flowering date (FFD) (ordinal day number) and Conrad's continentality index of individual tetraploid, hexaploid and pentaploid clones in the common garden in (a) 2009 and (b) 2010, and (c) difference in FFD between 2009 and 2010, where values are positive, flowering was later in 2010 than 2009.

APPENDIX S1

Supplementary information: methods and collectors

Methods

Collections

During field collections, leaf samples and stem cuttings were stored in polythene bags and kept cool for transport. Seeds were germinated and stem cuttings were rooted in a glasshouse for further study. Samples collected by volunteers were sent by post and analysed upon receipt.

Cytotype determination

Samples were analysed with either a Becton Dickinson FACSCalibur™ or a BD Accuri™ C6 flow cytometer with a red 488 nm wavelength laser, calibrated daily and run on the low flow rate (12 – 14 µl min^{-1}). Consistency between their outputs was checked by running duplicate samples. Analyses were repeated if full peak coefficients of variation exceeded 5 % or where DNA contents appeared to be outliers. To save resources, some samples were combined into groups of three. They were reanalysed separately if multiple peaks indicated mixed cytotypes.

Cx values

Samples from Britain were partly collected by volunteers who submitted them by post. We examined whether the time which elapsed between collection and processing and condition of the sample on receipt (fresh *vs.* desiccated; green *vs.* red/brown) affected the results. Desiccation and red/brown colouration both produced some erratic results and these data were excluded from Cx analyses.

Molecular investigations

Using universal primers (including those for microsatellites and other regions) (Demesure *et al.* 1995; Shaw *et al.* 2005; Weising and Gardner 1999), a subset of samples covering the range of cytotypes and full distribution of the collection were screened for variation in the chloroplast genome. Initial tests of seven primers for the chloroplast regions *trn*C-*trn*D, *mat*K, *trn*C-*ycf*6,

*trn*H-*trn*K, *trn*D-*trn*T, *ccmp*2 and *rbc*L were tested on a screening panel of eight samples. The primer sets for *trn*C-*trn*D and *mat*K failed to amplify during PCR, whilst the remaining five regions yielded a product. The successfully amplified regions were then tested on a larger panel of 24 samples and sequenced. Polymorphism was detected at all loci. Of these, *trn*C-*ycf*6, *trn*H-*trn*K, *trn*D-*trn*T were non-redundant and informative and were used to screen the full set of samples for variation.

Leaf tissue was taken from frozen samples. For each sample, roughly 1 cm^2 of tissue was ground to a fine powder using a Retsch Mixer Mill. DNA extraction was then carried out using QIAGEN DNeasy 96 Plant kits following the manufacturer’s protocol. Extracted DNA was assessed for quality and concentration on a 1% agarose gel before being frozen at -20 °C.

PCR amplification of all fragments was carried out in 25 µl reactions containing 2 µl genomic DNA, 200 µM each dNTP (Promega), 0.2 µM each primer (MWG Biotech), 2.5 µl of 10X PCR buffer (New England Biolabs), 0.5 U Taq DNA polymerase (New England Biolabs) and 1.6 % (v/v) bovine serum albumin. Reactions were prepared in 96-well plates and run on a Thermo MBS thermal cycler following published protocols (Demesure *et al.*, 1995) except for annealing temperatures of 62 °C for *trn*H-*trn*K, and 48 °C for *trn*D-*trn*T. Amplification of the *trn*C-*ycf*6 locus differed from the published protocol (Shaw *et al.*, 2005), with an initial denaturation step of 94 °C for three min, followed by 35 cycles of 94 °C, 55 °C and 72 °C each for 30s, and a final extension step of 72 °C for 10 min.

PCR products of *trn*C-*ycf6* and *trn*H-*trn*K were cleaned using 0.4 U Shrimp Alkaline Phosphatase (New England Biolabs) and 1 U Exonuclease I (New England Biolabs) to remove excess dNTPs and primers. Forward primers were added and the samples were then sent for sequencing at the NERC Biomolecular Analysis Facility sequencing service (Edinburgh Genomics) at the University of Edinburgh. Sequences were aligned using CodonCode Aligner (CodonCode Corporation) and manually checked.

Variation in the size of the *trn*D-*trn*T locus was assessed by running amplicons on 2 % agarose gel. A subset of samples was sequenced as above, and were found to contain large indels. Amplicons *c.* 800, 900, 1200 and 1300 bp were scored as 1, 2, 3, 4, respectively. All samples were scored by eye and characterised for their homology with one of the size categories.

References

Demesure B, Sodzi N, Petit RJ. 1995. A set of universal primers for amplification of polymorphic noncoding regions of mitochondrial and chloroplast DNA in plants. *Molecular Ecology* **4,** 129 - 131.

Shaw J, Lickey E, Beck J, *et al.* 2005. The tortoise and the hare II: relative utility of 21 noncoding chloroplast DNA sequences for phylogenetic analysis. *American Journal of Botany* **92,** 142 - 166.

Weising K, Gardener RC 1999. A set of conserved PCR primers for the analysis of simple sequence repeat polymorphisms in chloroplast genomes of dicotyledonous angiosperms. *Genome* **42**(1)**,** 9 -19.

Collectors

We thank all the people who collected leaf or seed samples for us. They are listed below.

Chris Andrews, Pedro Aphalo, Sheila Barker, Brian Ballinger, Beaver Creek Greenhouses, Botanic Garden (University of Bonn), Margaret Bradshaw, Stephen Bungard, Rebecca Chance, Rodney Cole, Ewan Cole, Rod Corner, Steve Cuttle, Mary Dean, Alison Donaldson, Edge of the Rockies Native Seed, Emorsgate Seeds, Ian Evans, Everwilde Seed, Jenny Farrar, Richard Friend, Gardens North (Canada), Diana Gilbert, John Grace, Alan Gray, Iain Gunn, Kevin Ingleby, Martin Jeffree, Megan Jeffree, Claire Kenna, Sanja Kovačić, Liz

Kungu, Annie Lamb, Elena Lapshina, Fiona Leckie, Tarja Lehto, Elizabeth Maddison, Hugh McAllister, Liz McDonnell, Barry & Jackie Metcalfe, Richard Milne, Ibby Moy, Munster Botanic Garden, staff of National Trust, staff of Natural England, Joy Newton, A. Peart, Anthony Taylor-Pigott, Jana Rauchová, Reykjavík Botanic Garden, Dave Riley, Linda Robinson, Royal Botanic Gardens Kew (Millenium Seedbank), Scotia Seeds, Lyn Selby, Paul Smith, Martin Steer, Julia Stephenson, Pauline Stokoe, students of Edinburgh University, students of University College Dublin, students of National University of Ireland at Galway, Audrey Summers, Tom Sunderland, David Sutton, Carrie Thomas, University Bayreuth Botanic Garden, Western Native Seed, Jill Williams, Graham Wilson, Mary Wilson, Wiltshire Wildlife Trust, Xinxin Xue.
